# Disabling Mitochondrial Peroxide Metabolism via Combinatorial Targeting of Peroxiredoxin 3 as an Effective Therapeutic Approach for Malignant Mesothelioma

**DOI:** 10.1371/journal.pone.0127310

**Published:** 2015-05-26

**Authors:** Brian Cunniff, Kheng Newick, Kimberly J. Nelson, Alexandra N. Wozniak, Stacie Beuschel, Bruce Leavitt, Anant Bhave, Kelly Butnor, Andreas Koenig, Edward T. Chouchani, Andrew M. James, Alexina C. Haynes, W. Todd Lowther, Michael P. Murphy, Arti Shukla, Nicholas H. Heintz

**Affiliations:** 1 University of Vermont, College of Medicine, Department of Pathology, 149 Beaumont Ave, Burlington, VT, 05405, United States of America; 2 University of Pennsylvania School of Medicine, Division of Pulmonary, Thoracic Oncology Research Laboratory, Philadelphia, PA, 19147, United States of America; 3 Wake Forest School of Medicine, Department of Biochemistry, Medical Center Boulevard, Winston-Salem, NC, 27157, United States of America; 4 University of Vermont, College of Medicine, Department of Surgery, 149 Beaumont Ave, Burlington, VT, 05405, United States of America; 5 University of Vermont, College of Medicine, Department of Radiology, 149 Beaumont Ave, Burlington, VT, 05405, United States of America; 6 University of Vermont, Department of Immunology medicine, 149 Beaumont Ave, Burlington, VT, 05405, United States of America; 7 Medical Research Council, Mitochondrial Biology Unit, Hills Road, Cambridge, CB2 0XY, United Kingdom; 8 Department of Medicine, University of Cambridge, Addenbrooke's Hospital, Hills Road, Cambridge, CB2 2QQ, United Kingdom; Instituto de Biociencias - Universidade de São Paulo, BRAZIL

## Abstract

Dysregulation of signaling pathways and energy metabolism in cancer cells enhances production of mitochondrial hydrogen peroxide that supports tumorigenesis through multiple mechanisms. To counteract the adverse effects of mitochondrial peroxide many solid tumor types up-regulate the mitochondrial thioredoxin reductase 2 - thioredoxin 2 (TRX2) - peroxiredoxin 3 (PRX3) antioxidant network. Using malignant mesothelioma cells as a model, we show that thiostrepton (TS) irreversibly disables PRX3 via covalent crosslinking of peroxidatic and resolving cysteine residues in homodimers, and that targeting the oxidoreductase TRX2 with the triphenylmethane gentian violet (GV) potentiates adduction by increasing levels of disulfide-bonded PRX3 dimers. Due to the fact that activity of the PRX3 catalytic cycle dictates the rate of adduction by TS, immortalized and primary human mesothelial cells are significantly less sensitive to both compounds. Moreover, stable knockdown of PRX3 reduces mesothelioma cell proliferation and sensitivity to TS. Expression of catalase in shPRX3 mesothelioma cells restores defects in cell proliferation but not sensitivity to TS. In a SCID mouse xenograft model of human mesothelioma, administration of TS and GV together reduced tumor burden more effectively than either agent alone. Because increased production of mitochondrial hydrogen peroxide is a common phenotype of malignant cells, and TS and GV are well tolerated in mammals, we propose that targeting PRX3 is a feasible redox-dependent strategy for managing mesothelioma and other intractable human malignancies.

## Introduction

Altered redox balance in tumor cells, characterized by an increase in the production of reactive oxygen species (ROS) and changes in antioxidant gene expression, supports a pro-proliferative state and evasion from apoptosis [1]. Increased oxidant production originates from multiple sources, including altered mitochondrial structure and function that leads to electron leakage that reacts with molecular oxygen forming superoxide radical [2,3]. The primary mitochondrial oxidant implicated in redox signaling is hydrogen peroxide (H_2_O_2_), which reacts with structurally distinct and solvent accessible low pKa cysteine residues on target proteins. Reversible oxidation of specific cysteine residues has been shown to modify the structure, function and subcellular distribution of numerous proteins [4].

Many proteins that are regulated via cysteine oxidation-reduction cycles, such as kinases, phosphatases and transcription factors, function in redox-responsive signaling circuits that control cell proliferation and survival [5]. Moderate levels of H_2_O_2_ support proliferation [6], while higher levels create a pro-oxidant environment leading to activation of stress response pathways, damage of cellular macromolecules and cell death [7]. Due to oncogene activation and changes in cellular metabolism, neoplastic transformation results in a pro-oxidative state that may induce cell cycle arrest, cellular senescence or apoptosis [8]. Tumor cells escape from redox-dependent cytotoxic responses via loss of tumor suppressor genes and/or up-regulation of antioxidant enzymes and stress response factors, allowing tumor cells to prosper in a pro-oxidative state [9]. Because this phenotypic adaptation is not limited to a specific subset of oncogenes and tumor suppressor genes, exploiting perturbations in the metabolism of mitochondrial and cytosolic-derived oxidants has been proposed to be a viable therapeutic target in a variety of human cancers [10,11].

Altered oxygen metabolism in cancer cells has been evident since the seminal studies of Otto Warburg [12]. The preference for glycolysis, even under aerobic conditions, fostered the belief that mitochondria were damaged in tumor cells. Mutations in mitochondrial DNA do indeed promote tumorigenesis [13], but mitochondria from tumor cells generally have only subtle alterations in energy transfer [14,15]. Rather, cancer cells reorganize their metabolic machinery in response to an imbalanced redox status that originates from rapid growth, changes in oxygen tension and low nutrient availability [16]. Mitochondrial reserve capacity, which is the difference between maximal and basal respiration, has been shown to play an important role in cell tolerance to changes in ROS levels [17,18]. Mitochondria from tumor cells have reduced reserve capacity and cannot tolerate excessive ROS production as efficiently as normal cell mitochondria [19]. There is considerable interest in exploiting these features of metabolic vulnerability for therapeutic intervention.

The antioxidant network composed of NADPH, thioredoxin reductase 2 (TR2), thioredoxin 2 (TRX2) and peroxiredoxin 3 (PRX3) is the primary system responsible for metabolism of mitochondrial H_2_O_2_ [20]. PRX3, which is found exclusively in the mitochondrial matrix [21], is a member of the typical 2-Cys peroxiredoxin family (PRX 1–4). 2-Cys PRXs metabolize hydroperoxides in a multistep process that involves oxidation of a peroxidatic cysteine to sulfenic acid (–SOH), spontaneous disulfide bond formation with a resolving cysteine located on the adjacent PRX subunit (i.e. forming PRX-S-S-PRX), and subsequent reduction by the oxidoreductase TRX to regenerate active enzyme [22]. Elevated expression of PRX3 is linked to resistance to apoptosis and increased cell proliferation [23,24]. PRX3 is over-expressed in multiple cancers [25], and increased expression may be related to adaptive responses required to maintain mitochondrial function. For example, PRX3 expression in MCF-7 and MDA-MB-231 breast cancer cells promotes cell cycle progression, while silencing PRX3 impairs cell proliferation [24]. Prostate cancer cells over-expressing PRX3 also grow faster than their control counterparts [26].

Recently, mitochondrial oxidants were shown to be essential for tumorigenesis mediated by activated K-RAS [10], which induces ROS-dependent cell senescence in normal cells [8]. Interestingly, the pro-oxidant state induced in mitochondria by activated K-RAS is counterbalanced through increased expression of Forkhead Box M1 (FOXM1), a redox-responsive transcription factor that promotes expression of the mitochondrial antioxidant enzymes manganese superoxide dismutase (MnSOD or SOD2) and PRX3, permitting cells to escape from ROS-induced senescence [27].

Thiostrepton (TS) is a thiazole antibiotic that has shown promise as a cancer therapeutic, specifically through targeting FOXM1 [28,29] and induction of oxidative and proteotoxic stress [30]. Fragments of TS have been reported to directly bind FOXM1 in human breast MCF-7 cells, blocking the recruitment of FOXM1 to target promoter sites [31]. Others have proposed TS acts as a proteasome inhibitor [32], though this effect may be secondary to the induction of oxidative stress [30]. TS has also been shown to sensitize melanoma cells to growth inhibition by arsenic trioxide through an ROS-dependent mechanism [33].

We recently identified PRX3 as a redox-dependent target of TS in malignant mesothelioma (MM) cells [34]. Treatment of MM cells with TS leads to stable, non-reducible and irreversible modification to PRX3, inhibits expression of FOXM1, increases mitochondrial oxidant levels, hyperactivates ERK1/2 and induces cell death, all in a redox-dependent manner [34]. Mitochondrial TRX2 is the oxidoreductase responsible for reduction of PRX3 disulfide-bonded dimers, and co-treatment of MM cells with the triphenylmethane gentian violet (GV), a TRX2 inhibitor [35], markedly potentiates modification of PRX3 by TS.

Here we describe a model for the molecular mechanism of TS that exploits altered oxidant metabolism in the mitochondria of malignant mesothelioma cells. We propose adduction of specific cysteine residues in PRX3 by TS inactivates its peroxidase activity, thereby compromising adaptive responses that permit mesothelioma cells to tolerate a pro-oxidant state. By inducing the accumulation of PRX3-S-S-PRX3 dimers, we propose GV increases adduction of the neighboring catalytic site that is locally unfolded [36]. Evaluation of the effects of TS and GV, alone or together, in a SCID mouse xenograft model of human MM indicates combinatorial targeting of the PRX3 antioxidant network is a feasible strategy for managing a wide variety of tumors characterized by dysregulation of mitochondrial metabolism that results in high oxidant production.

## Results

### Thiostrepton irreversibly modifies PRX3 and increases mitochondrial H_2_O_2_ levels

**Fig 1:**
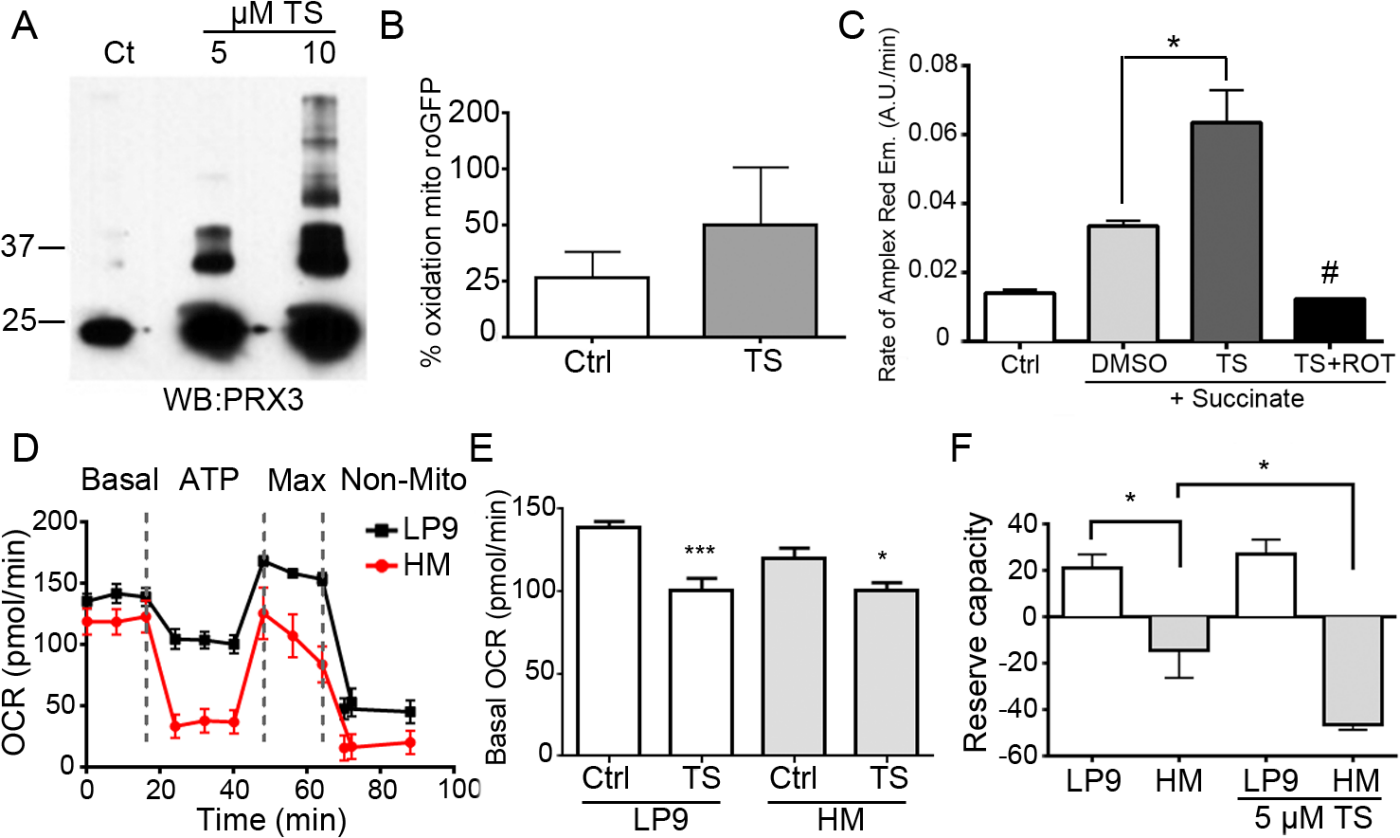
Thiostrepton modifies PRX3, increases mitochondrial H_2_O_2_ and disrupts mitochondrial bioenergetics in MM cells. (A) Recombinant PRX3 (rPRX3) was incubated with indicated concentration of thiostrepton (TS) for 30 min and visualized by immunoblotting with anti-PRX3 antibody after separation by reducing SDS-PAGE. (B) Mitochondrial redox status was analyzed by ratiometric live cell imaging of mito-roGFP in HM cells treated with 5 μM TS for 6 hr (n = 10 cells). (C) Hydrogen peroxide production in isolated mitochondria respiring on succinate (+ succinate) in the presence of indicated compounds (TS = thiostrepton, ROT = rotenone) (* p < 0.05, # p < 0.05 compared to TS). (D) Bioenergetic profiles (oxygen consumption rate, OCR) for LP9 and HM cells (n = 5). Dotted lines indicate time points of drug injections (oligomycin, CCCP, rotenone/antimycin A, respectively) (E) Basal OCR of LP9 and HM cells treated with 5 μM TS for 6 hr (n = 5, * p < 0.05, *** p < 0.001, Error bars represent SEM). (F) Reserve capacity of LP9 and HM cells untreated or treated with 5 μM TS for 6 hr (n = 5, * p < 0.05, Error bars represent SEM). See also S2 Fig.

Previously our group showed that the thiazole antibiotic thiostrepton (TS) irreversibly modifies PRX3 in MM cells in a redox-dependent manner [34]. As before, treatment of recombinant PRX3 (rPRX3) with TS resulted in the dose-dependent formation of rPRX3 species with retarded electrophoretic mobility on reducing SDS-PAGE (Fig 1A, lanes 2–3). Modification of rPRX3 was greatly diminished when rPRX3 was not fully reduced prior to reaction with TS (data not shown). The modified rPRX3 species were resistant to reduction by DTT and TCEP, and at 5 μM TS rPRX3 migrated with an apparent molecular weight of ~35–40 kDa (Fig 1A). Under reducing and denaturing conditions cellular PRX3 migrates as ~23 kDa monomers [20], but in extracts from cells treated with TS modified PRX3 migrates at ~35–40 kDa, the apparent molecular weight of PRX3 homodimers [20]. Since higher concentrations of TS resulted in the formation of additional species of higher molecular weight (Fig 1A), other non-reducible oligomers of PRX3 are also possible. Immunoprecipitation of PRX3 dimers and monomers from extracts of HM cells treated with TS and subsequent analysis of tryptic peptides confirmed that the modified immunoreactive PRX3 species migrating at 35–40 kDa contained PRX3 peptides (S1A and S1B Fig).

To test the effect of TS on the oxidation state of cellular mitochondria, HM cells were transfected with an expression vector for mito-roGFP and ratiometric imaging was used to measure mitochondrial redox status. Treatment of cells with 5 μM TS for 6 hr tended to shift mitochondria to a more oxidized environment (Fig 1B). In support of this observation, purified mitochondria treated with TS produced more hydrogen peroxide *in vitro* (Fig 1C). Isolated rat heart mitochondria were incubated with succinate to induce reverse electron transport (RET), which leads to H_2_O_2_ production from electron transport chain complex I [37]. Addition of TS to mitochondria respiring on succinate led to an increase in H_2_O_2_ production as compared to DMSO controls, and this increase was completely blocked by the complex I inhibitor rotenone (Fig 1C), which blocks RET and reduces H_2_O_2_ production [38].

Using extracellular flux analysis, the effects of TS on the oxygen consumption rate (OCR) and media acidification were measured in HM and hTERT immortalized LP9 human mesothelial cells (Fig 1D, S2A and S2B Fig). Basal OCRs were very similar between the two cell types, but addition of the mitochondrial ATP synthase inhibitor oligomycin reduced the OCR in LP9 cells to a much lesser extent than in HM cells (Fig 1D and 1E), indicating LP9 mesothelial cells have a lower demand for ATP [39]. Addition of the proton ionophore CCCP was used to uncouple electron transport from the proton gradient and quantify the maximal mitochondrial respiration rate, as the difference between the maximal respiration and basal respiration rate represents mitochondrial reserve capacity. As compared to LP9 cells, HM cells had virtually no reserve capacity, and TS reduced this limited reserve capacity to a higher extent in HM cells than LP9 cells (Fig 1D–1F). TS reduced the basal OCR to nearly the same extent in LP9 and HM cells (Fig 1E); TS had no significant effect on the extracellular acidification rate (S2C and S2D Fig). Cumulatively these data show that TS covalently modifies PRX3, inhibits basal oxygen consumption, increases the intra-organelle oxidation state of mitochondria and increases mitochondrial production of H_2_O_2_.

### Disulfide-bonded PRX3 may be the preferred target for TS

**Fig 2:**
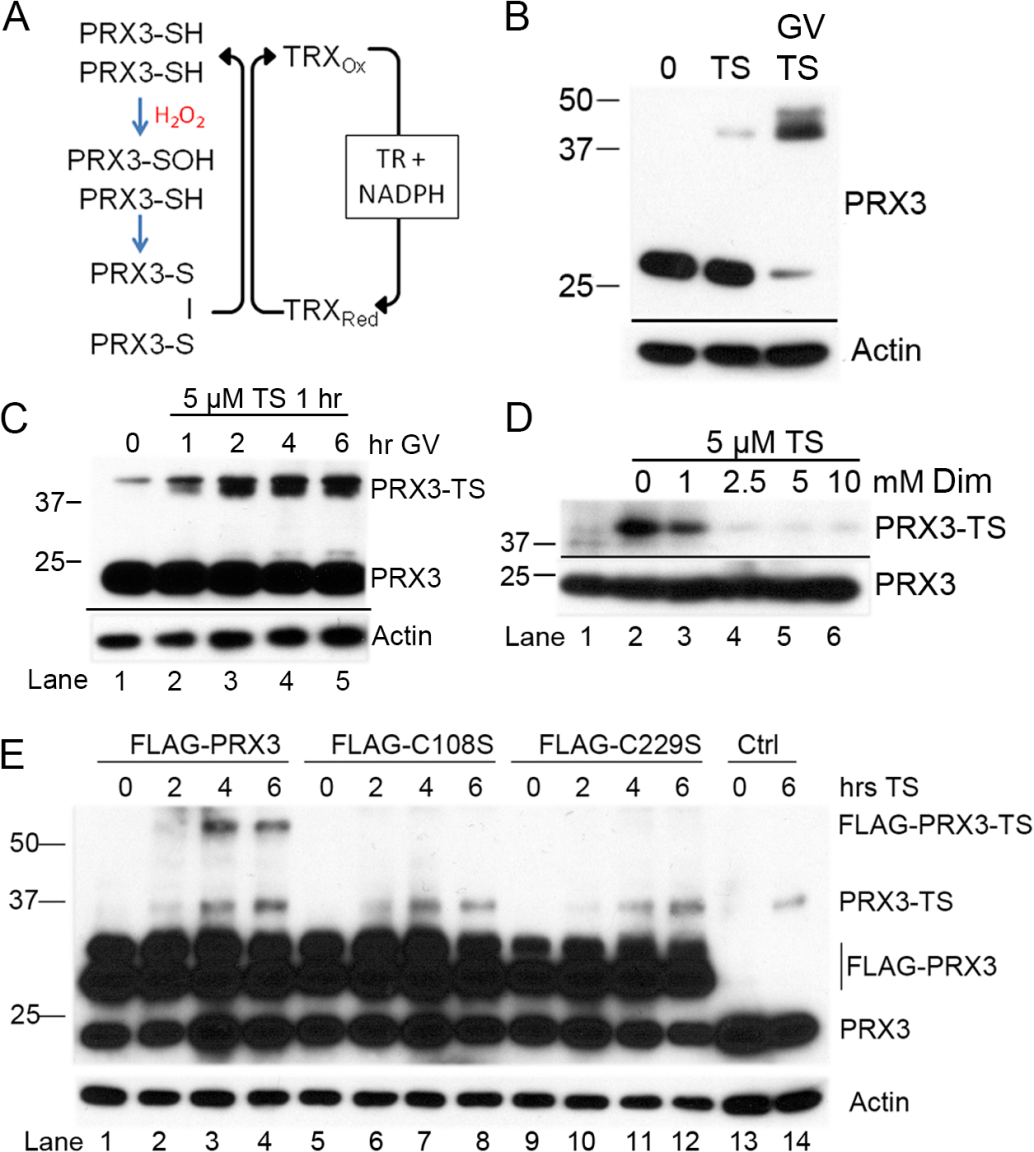
PRX3 turnover promotes adduction of specific cysteine residues by thiostrepton in cells. (A) Reconstitution of the PRX3 catalytic cycle *in vitro* with purified components. (B) MM cells were treated with 5 μM TS for 18 hr or pre-incubated with 1 μM GV for 6 hr then treated with 5 μM TS (G/T) for 18 hr and immunoblotted for PRX3 after reducing SDS-PAGE. (C) MM cells were pre-incubated with 1 μM GV for the indicated times and then treated with 5 μM TS for 1 hr and cell lysates were immunoblotted for PRX3 after reducing SDS-PAGE. (D) Pre-incubation of MM cells with dimedone (Dim) for 6 hr blocked TS induced modification of PRX3. (E) HM Cells transfected with Flag-Tagged PRX3 expression plasmids were treated with 5 μM TS, lysates were collected at the indicated time points and TS induced modifications of PRX3 were visualized by immunoblotting with anti-PRX3 antibody after separation by reducing SDS-PAGE. See also S1 Fig.

PRX3 functions as head-to-tail homodimers that can assemble into dodecamers [40,41], and is actively recycled after oxidation of the peroxidatic cysteine in a multi-step process that requires reduction of the disulfide bond between opposing monomers by TRX2, and subsequent reduction of TRX2 by thioredoxin reductase 2 (TR2) using reducing equivalents from NADPH (Fig 2A).

We first investigated if an active catalytic cycle is required for modification of PRX3 by TS in HM cells. Treatment of HM cells with TS for 18 hr resulted in formation of the ~35–40 kDa modified PRX3 species, and this modified species was markedly enhanced by pre-incubating cells with GV for 6 hr prior to exposure to TS (Fig 2B). We then tested the effect of pre-incubating cells with GV for different times on the degree of adduction of PRX3 by TS. As shown in Fig 2C, longer pre-incubation times increased the degree of adduction by a fixed concentration of TS in 1 hr. Moreover, as oxidation of the peroxidatic cysteine to sulfenic acid (-SOH) is the first step in catalysis, HM cells were incubated for 6 hr with dimedone, a compound which specifically reacts with sulfenic acids [42], and then treated with TS. Dimedone blocked the formation of the modified species of PRX3 in a dose-dependent fashion (Fig 2D, lanes 2–6), indicating that an active catalytic cycle markedly promotes adduction of PRX3 by TS.

**Fig 3:**
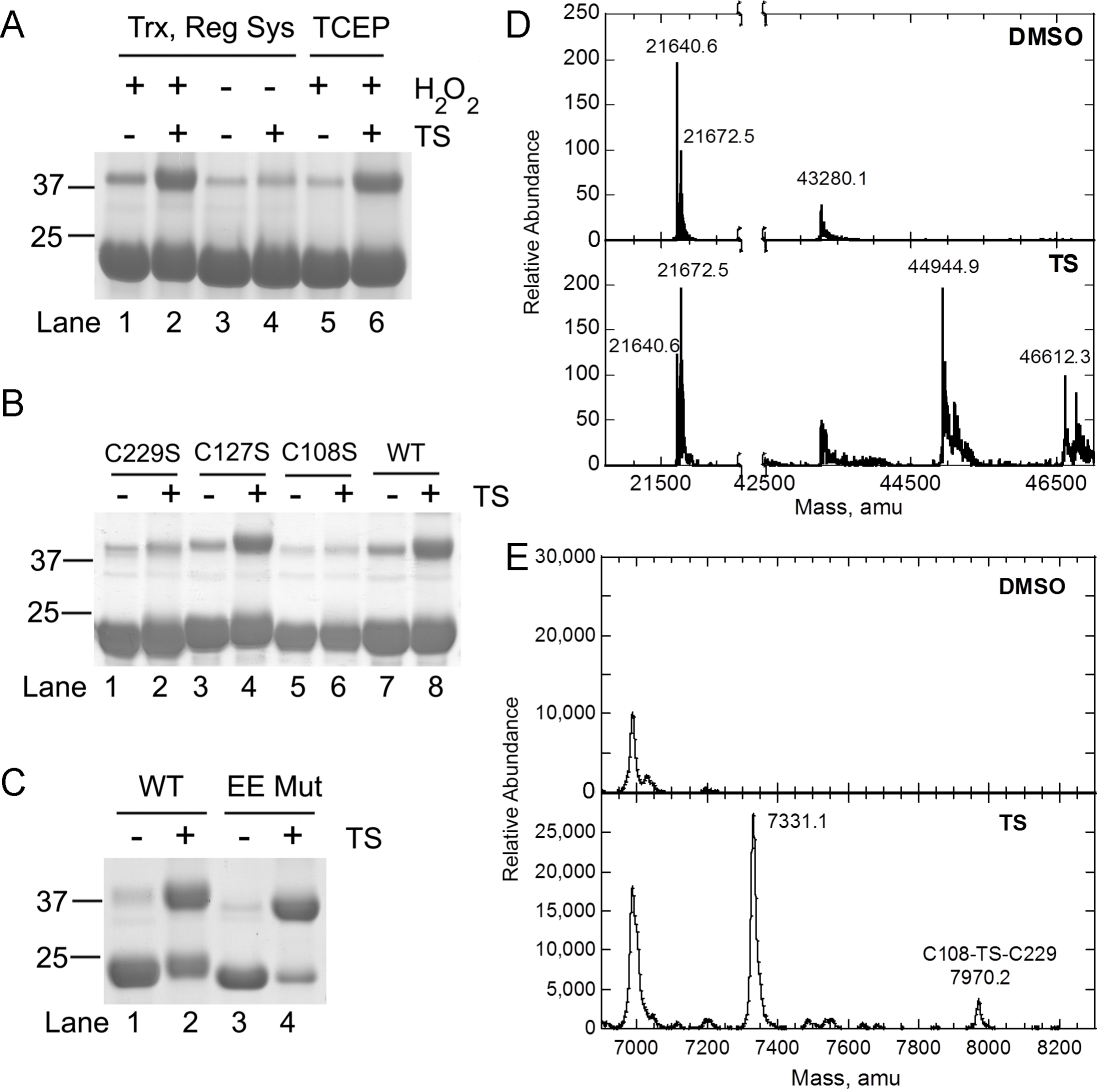
TS adducts specific cysteines of rPRX3 *in vitro*. (A) *In vitro* reaction between recombinant wild type (WT) PRX3 (100 μM) and 200 μM TS. Reactions contained either the Trx regeneration system or TCEP. The samples were treated with 6 successive additions of 100 μM H_**2**_O_**2**_ and 200 μM TS, as indicated. Samples were resolved by reducing SDS-PAGE and visualized by staining for total protein with GelCode Blue. (B) WT PRX3 or the indicated PRX3 mutants were incubated with or without TS as in panel A and formation of irreducible PRX3 dimers was visualized by staining for total protein after reducing SDS-PAGE. (C) WT PRX3 and the dimeric variant of PRX3 (EE Mut) were treated with 200 μM TS. (D) MS analysis of TS adducts. The EE Mut samples shown in lanes 3 and 4 of panel C were treated with 20 mM DTT to reduce disulfide bonds and to block further reactions of thiostrepton dehydroalanines with PRX3 thiols. The molecular weight of the resulting PRX3 adducts were determined by ESI-TOF MS. The signals at 21640.6 and 21672.5 atomic mass units (amu) correspond to the average molecular weight of the reduced monomer (-SH, 21640.6 amu) and sulfinic acid monomer (-SO_**2**_, 21672.6 amu), respectively. The peak at 43280.1 amu corresponds to a PRX3 dimer containing one disulfide (theoretical MW = 43,278.2) that presumably occurs as a result of trace amounts of oxidants present during buffer exchange prior to MS analysis. The signals at 44,944.9 and 46,612.3 amu correspond to 2 PRX3 monomers linked by 1 (44,945.0 amu) and 2 (46,609.8 amu) thiostrepton adducts, respectively. (E) Samples from (D) were digested with trypsin and peptides were analyzed by MALDI-TOF MS. The peptide at 7970.2 amu corresponds to a single thiostrepton linked to both the C108 and C229 peptides in PRX3 (7971.0 amu).

Reconstitution of the PRX3 catalytic cycle with purified components in an *in vitro* PRX3 turnover assay (See materials and methods) also supported the possibility that an active catalytic cycle promotes adduction by TS. Human recombinant PRX3 (rPRX3), *E*. *coli* TRX2, and *E*. *coli* TR were incubated with an NADPH regenerating system and the reactions were pulsed with H_2_O_2_ in the presence or absence of TS. Pulsing the reconstituted system with H_2_O_2_ was intended to induce rPRX3 oxidation and regeneration (referred to as “PRX3 turnover”), but a priori does not reproduce the physiological flux of H_2_O_2_ in cellular mitochondria. During active cycling in the presence of TS, a significant amount of PRX3 was converted to the non-reducible dimer adduct. (Fig 3A, lanes 1 and 2). Although similar levels of adduct was formed when the non-thiol reductant TCEP was used (Fig 3A, lanes 5 and 6), minimal dimer was observed when H_2_O_2_ was not present and the PRX3 was not allowed to cycle (Fig 3A, lanes 3 and 4).

### TS adducts specific cysteine residues in PRX3

TS has been reported to bind to prokaryotic ribosomes and inhibit protein synthesis [43]. TS contains three dehydroalanine residues which can form a Michael addition product with cysteine residues and other thiols to generate non-reducible thioethers [44,45]. A single TS has been shown to covalently adduct cysteine residues in the bacterial transcription factor TipAS through dehydroalanine moieties, and does not react with any other free amino acid other than cysteine [46]. Human mitochondrial PRX3 contains three cysteine residues: the peroxidatic cysteine at position 108 (Cys108), the resolving cysteine at position 229 (Cys229), and a highly conserved but non-catalytic cysteine at position 127 (Cys127). Preliminary studies confirmed that TS reacts with reduced thiols such as reduced glutathione (GSH) and N-acetyl-L-cysteine (NAC) (S1C and S1D Fig) to some degree, but not oxidized glutathione (GSSG, data not shown).

We next investigated TS-induced modifications to rPRX3 mutants where specific cysteine residues corresponding to Cys108, Cys229 and Cys127 were replaced with serine. In the full rPRX3 catalytic system addition of TS induced modifications to wild type rPRX3 as expected (Fig 3B, lanes 7–8). Incubation of the Cys108 and Cys229 serine mutants significantly reduced the levels of modification to rPRX3 by TS (Fig 3B, lanes 1–2 and 5–6), whereas the Cys127 mutant showed TS induced modifications equal to that of wild type PRX3 (Fig 3B lanes 3–4).

In order to test the hypothesis that the dimeric form of PRX3 is more reactive with TS, we utilized an engineered dimer of PRX3, S139E/A142E (EE Mut). This variant is unable to form high molecular weight (HMW) species (i.e. decamer or dodecamers) due to the introduction of two negatively charged residues into the dimer-dimer interface. The amount of non-reducible dimer formed upon addition of TS was greater in the EE mutant than in WT Prx3 (Fig 3C), indicating that HMW oligomer formation is not required to form the TS adduct and that the TS adduct does not form across the dimer-dimer interface.

ESI-TOF MS was performed on intact EE mutant that had been cycled in the presence or absence of TS and then subsequently treated with DTT to reduce disulfides and inactivate any remaining TS. In both samples, peaks were observed at 21,640.6 and 21,672.5 atomic mass units (amu) for the reduced (-SH, theoretical average MW = 21,640.6) and hyperoxidized (-SO_2_H, theoretical MW = 21,672.8) monomer (Fig 3D and S1 Table). In the presence of TS, two new peaks are observed at 44,944.9 and 46,612.3 amu that correspond to two Prx3 monomers linked by either one or two TS molecules (theoretical MW = 44,945.0 and 46,609.8, respectively, Fig 3D).

The EE samples utilized for ESI-MS above were digested with trypsin and the resulting peptide mixture was analyzed by MALDI-TOF MS analysis. Peptides were observed in the TS-treated sample that agreed with the predicted molecular weight for single TS adducts with Cys127 and Cys229 (S1 Table). Importantly, we were able to directly observe a peptide in the TS sample that corresponds to a single TS molecule linked to both the Cys108 and Cys229 containing peptides (Fig 3E, obs MW = 7970.2, predicted MW = 7971.0); this peptide-TS complex was not observed in the DMSO control. This data supports that TS is able to irreversibly react with all three cysteines in Prx3, but that the irreducible dimer formation occurs through the reaction of TS with Cys108 and Cys229.

To examine adduction of PRX3 further, wild type and cysteine mutants of FLAG-tagged human PRX3 were expressed in HM cells (Fig 2E). In these expression constructs the FLAG tag is located on the amino terminus of the mature protein downstream of the mitochondrial targeting sequence (M. Hampton, personal communication). Immunoblotting of extracts of transfected HM cells with PRX3 antibody revealed two bands that likely represent the processed species located in mitochondria and the larger uncleaved cytoplasmic precursor (Fig 2E, lanes 1–12). Treatment of transfected cells with TS resulted in the formation of non-reducible species of PRX3 with the expected mobility of endogenous PRX3 and FLAG-PRX3 dimers (Fig 2E, lanes 2–4). FLAG-tagged PRX3 lacking the Cys108 (lanes 5–8), and Cys229 (lanes 9–12) were not modified by TS, although the TS-adduct was observed for endogenous PRX3 (lanes 1–4). Altogether, the data from the analysis of Cys variants of PRX3, either as recombinant proteins or cellular expression constructs, supports the conclusion that an active PRX3 catalytic cycle is required. Moreover, TS-adduct formation with the PRX3 dimer can crosslink Cys108 and Cys229. The need for a fully functional catalytic cycle to form an intermediate that is highly susceptible to TS adduction is further supported by the increase in adduct formation when the activity of TRX2 is inhibited by GV (Fig 2B and 2C).

### PRX3 modification by TS correlates with cell viability

**Fig 4:**
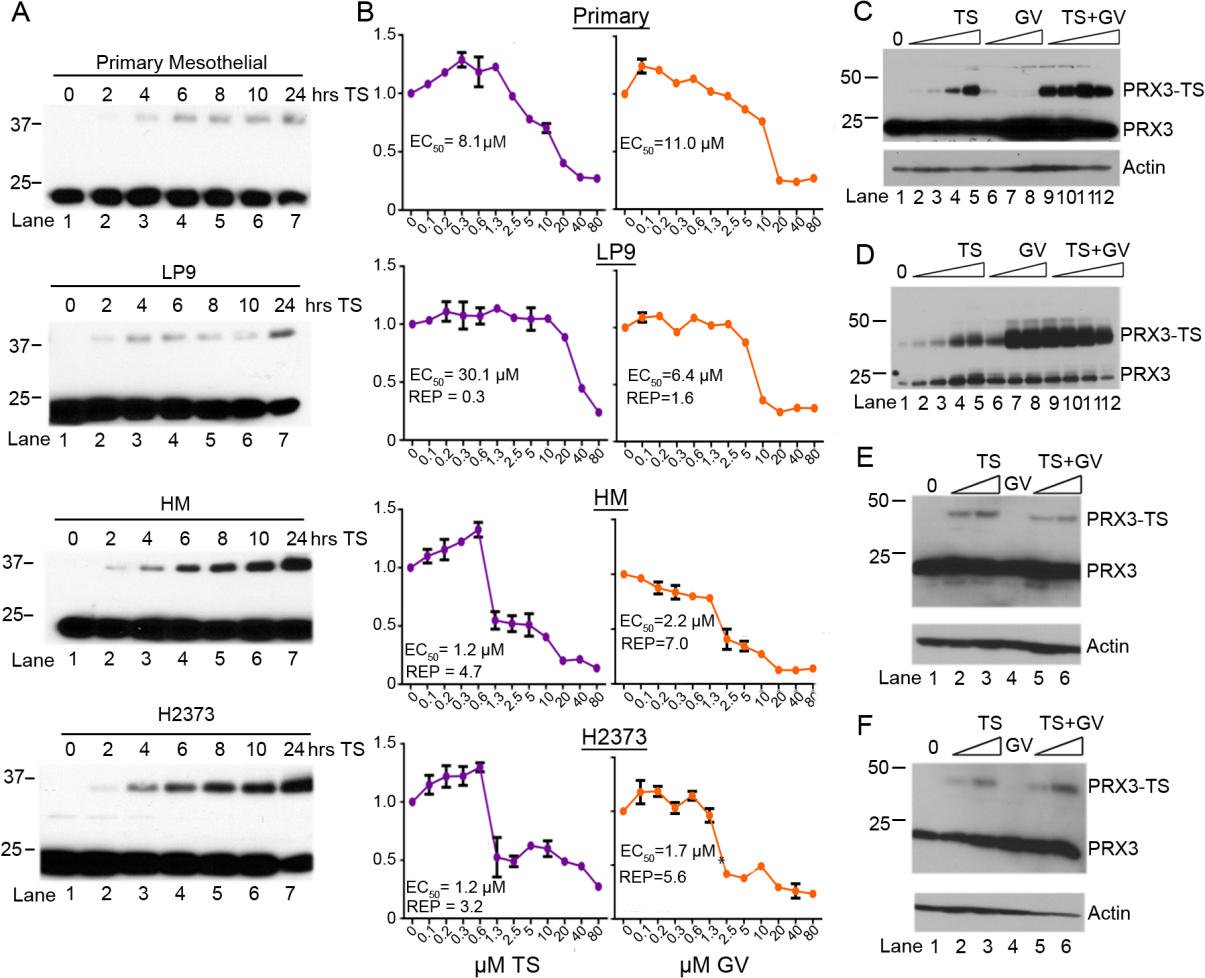
Adduction of PRX3 by TS correlates with cytotoxicity. (A) Human primary mesothelial, immortalized LP9 mesothelial, and HM and H2373 mesothelioma cell lines were incubated with 5 μM TS and lysates were collected at indicated time points over 24 hr. Formation of TS induced PRX3 dimers was visualized by reducing SDS-PAGE and immunoblotting with anti-PRX3 antibody. (B) Cell lines from (A) were incubated with indicated concentrations of TS (left) or GV (right) for 24 hr and total cell mass was determined by staining with crystal violet (Y axis values were normalized to untreated cells). The EC_**50**_ values for the indicated cell lines to TS or GV and the relative potency (REP) of TS and GV, as compared to primary mesothelial cells, are shown. (C) HM cells were treated with increasing concentrations of TS, GV, or TS + GV for 18 hr and extracts were resolved by reducing SDS-PAGE. PRX3 modification was assessed by immunoblotting as before. Note that GV accentuates modification of PRX3 by TS by blocking the activity of TRX2. (D) Extracts from cells treated as in panel C were resolved by non-reducing SDS-PAGE and PRX3 monomers (~23 kD) and disulfide-bonded dimers (~46 kD) were assessed by immunoblotting for PRX3. Note that GV markedly increased the level of disulfide-bonded PRX3 dimers, an indication of severe mitochondrial oxidative stress. (E) HMC2 and (F) HMC3 human primary mesothelial cell cultures were incubated with increasing doses of TS, GV or TS + GV for 18 hours, and assessed for PRX3 expression after reducing SDS-PAGE by immunoblotting. The formation of the modified species of PRX3 in response to TS was less evident in primary mesothelial cells.

PRX3 is over-expressed in mesotheliomas, though it is not considered a prognostic factor [47]. Surgical specimens from the pleural wall of human patients showed low levels of expression of PRX3, but once propagated in culture, human primary mesothelial cells (HMCs) showed little difference in expression of PRX3 as compared to immortalized LP9 mesothelial cells and MM cell lines (data not shown). To examine the sensitivity of PRX3 to adduction by TS, passage 3 primary human mesothelial cells (HMCs), LP9 immortalized but non-tumorigenic mesothelial cells, and HM and H2373 MM cell lines were incubated with 5 μM TS and cell lysates were prepared over 24 hr. The degree of modification of PRX3 by TS was markedly higher in MM cell lines, as compared to primary or immortalized mesothelial cells (Fig 4A). Quantification of total cell mass with crystal violet staining showed significant dose-dependent differences in the cytotoxicity of TS between normal mesothelial and MM cells (Fig 4B, left panel). The EC_50_ of TS in HM and H2373 MM cells was 1.2 μM, ~7 times lower than primary HMCs with an EC_50_ of 8.1 μM and ~25 times lower than that observed with immortalized LP9 mesothelial cells (EC_50_ = 30.1 μM). Similarly primary human mesothelial cells were less sensitive to GV as compared to human HM and H2373 MM cells (Fig 4B, right panel). Unlike TS, GV does not irreversibly modify PRX3, as under denaturing and reducing conditions PRX3 from HM cells treated with GV migrated in SDS polyacrylamide gels as reduced and/or hyperoxidized monomers at ~23 kD (Fig 4C, lanes 6–8). However, under non-reducing conditions that preserve disulfide bonds, GV induced the marked accumulation of disulfide-bonded PRX3 dimers (Fig 4D, lanes 6–8), and under these conditions GV potentiates the adduction of PRX3 by TS (Fig 4D, lanes 9–12). In two independent isolates of human primary mesothelial cells (HMC2 and HMC3), TS induced much lower levels of modification of PRX3, and these levels were not enhanced by GV (Fig 4E and 4F). Based on observations that indicate MM tumor cells produce more mitochondrial oxidants, have a more oxidized mitochondrial environment and have no respiratory reserve capacity (Fig 1B and 1F), which are all common properties of human tumor cells, we conclude that the toxicity of TS and GV are enhanced in MM tumor cells by constitutively higher demand for detoxification of H_2_O_2_ by the TR2-TRX2-PRX3 antioxidant network.

### shPRX3 MM cells are less sensitive to TS than WT controls

**Fig 5:**
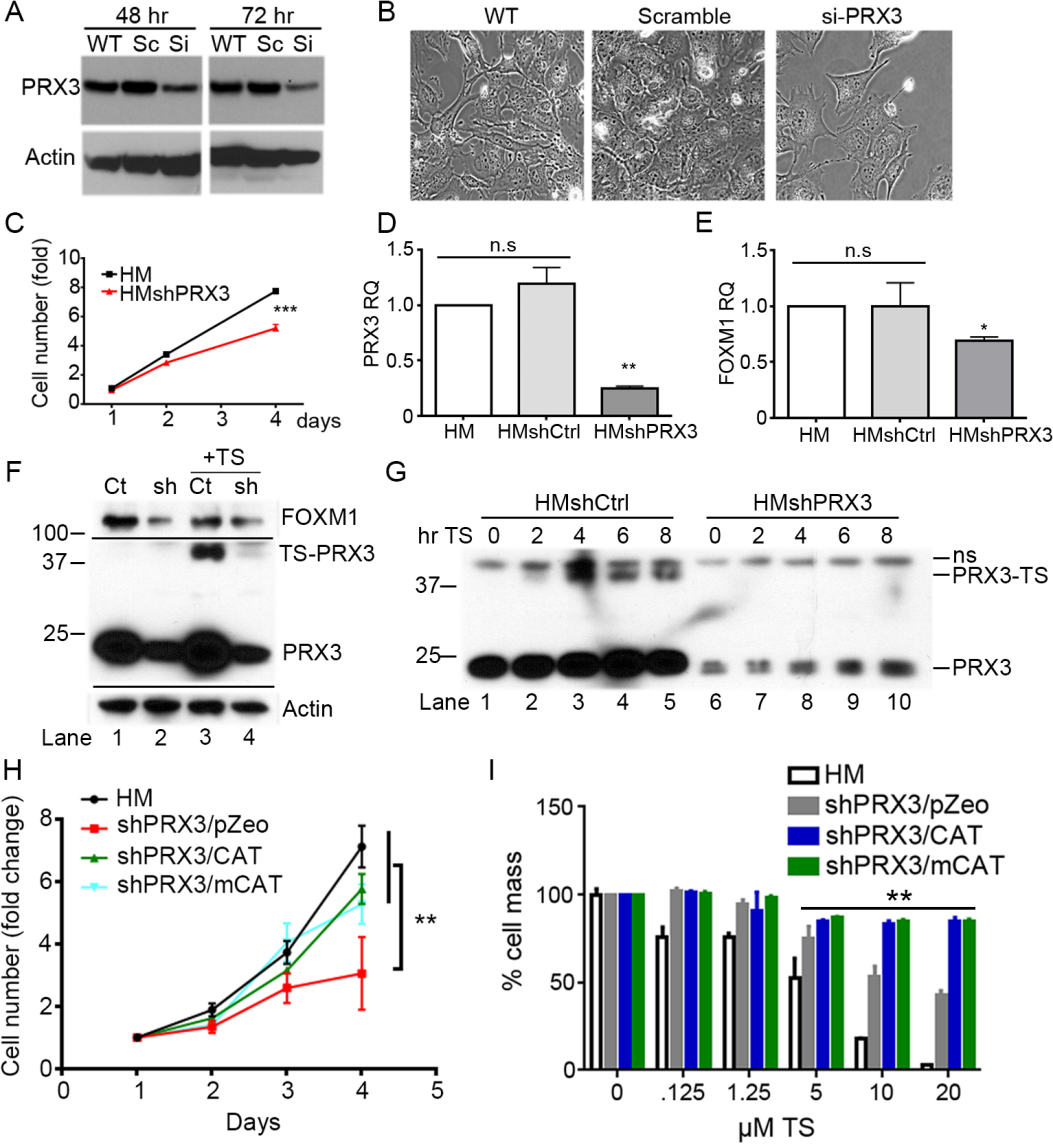
shPRX3 cells are less sensitive to TS than WT MM cells. (A) PRX3 expression in cells treated with scrambled control (Sc) or PRX3 siRNA for 48 and 72 hr. (B) Cell morphology and density of cells as treated in panel A after 72 hr. (C) Cell number in HMshPRX3 cells as compared to controls over 4 days (n = 4, *** p < 0.001). (D and E) Transcript levels for PRX3 and FOXM1 in WT HM, HMshCtrl cells and HMshPRX3 cell lines (** p < 0.01, * p < 0.05, n.s. = not significant). (F) Cell lysates from HMshCtrl and HMshPRX3 cell lines incubated with 5 μM TS for 18 hr were examined for PRX3, FOXM1, and actin expression by immunoblotting after denaturing SDS-PAGE. (G) HMshCTRL and HMshPRX3 cells were treated with 5 μM TS, lysates were collected over time and examined for PRX3 by immunoblotting (ns = non-specific band). (H) Cell number in shPRX3/pZeo, shPRX3/CAT, and shPRX3/mCAT cells compared to HM controls measured over 4 days (n = 4), ** p < 0.01) (I) HM controls, shPRX3/pZeo, shPRX3/CAT, and shPRX3/mCAT cells were incubated with increasing concentrations of TS for 18 hr, and total cell mass was determined by staining with crystal violet (n = 4, ** statistically significant all groups compared to HM, p <0.01). Error bars represent SEM. See also S3 Fig.

To ascertain if PRX3 is an important primary target of TS, RNA interference was used to knock-down expression of PRX3. In transient transfection experiments with HM cells siRNA targeted to PRX3 mRNA reduced PRX3 protein expression 3–5 fold, whereas the scrambled siRNA control had no effect (Fig 5A). Transfection of HM cells with siRNA to PRX3 resulted in lower cell density (Fig 5B), and stable expression of shRNA to PRX3 reduced HM and H2373 MM cell proliferation as compared to vector controls (Fig 5C and S3A Fig), as has been reported for breast cancer cells [24]. Interestingly, in addition to inhibition of expression of PRX3 mRNA (Fig 5D and S3B Fig) and protein (Fig 5F), shPRX3 cells also showed lower expression levels of FOXM1 mRNA (Fig 5E and S3D Fig). Comparison of wild type or control HM cells to shPRX3 knock-down cells by immunofluorescence microscopy (S3E–S3G Fig) showed that inhibition of PRX3 expression resulted in lower levels of both cytoplasmic and nuclear isoforms of FOXM1. Reduced FOXM1 expression in shPRX3 cells as compared to vector controls was evident by immunoblotting (Fig 5F, lanes 1 and 2), and adduction of PRX3 and inhibition of FOXM1 expression by TS was also reduced in shPRX3 cells (Fig 5F, lanes 3–4).

To further investigate the sensitivity of cells with reduced PRX3 expression to TS, vector control (HMshCtrtl) and shPRX3 (HMshPRX3) cells were treated with TS for different time periods and the levels of the 35–40 kDa-modified species were evaluated by immunoblotting (Fig 5G). In shPRX3 cells the modified form of PRX3 failed to accumulate over time (Fig 5G, lanes 6–10); this result may be due to reduced levels of PRX3 and/or reduced levels of disulfide-bonded PRX3 intermediates in the PRX3 catalytic cycle.

Due to the primary role of PRX3 in metabolizing mitochondrially-derived H_2_O_2_ we investigated whether expressing the H_2_O_2_ scavenger catalase or catalase targeted to mitochondria (mito-catalase) in shPRX3 cells could rescue the proliferation defects shown in Fig 5H. As indicated above shPRX3 cells (shPRX3/pZeo) grew significantly slower than HM controls and this defect in cell proliferation was rescued by the stable expression of catalase (shPRX3/Cat) or mito-catalase (shPRX3/mCat). Since HM cells stably expressing shRNAs to PRX3 showed significantly less modification to PRX3 after TS treatment (Fig 5G), we evaluated the dose response of shPRX3 cells to TS treatment. HM cells expressing shRNAs to PRX3 were significantly less sensitive to increasing concentrations of TS while shPRX3 cells expressing catalase or mito-catalase showed further reduction in sensitivity to TS (Fig 5I). These data collectively support the conclusion that PRX3 is a relevant and specific target for TS and cellular redox status influences TS bioactivity.

### TS and GV impede tumor progression in a SCID mouse xenograft model of MM

The *in vivo* efficacy of TS and GV has been independently investigated in solid tumor models with promising results [48,49]. We confirmed these findings using a subcutaneous xenograft model in Fox Chase SCID mice. Administration of 5 mg/kg TS every other day impaired tumor growth and reduced FOXM1 expression (S4 Fig). However, subcutaneous tumor burdens are irrelevant in MM as the primary lesions arise in the pleural and peritoneal cavities. To test the effects of TS and GV in the peritoneal cavity, Fox Chase SCID mice were injected intraperitoneally (IP) with 2 to 5 x 10^6^ HM cells and tumors were allowed to become established for two weeks. Six mice were randomly assigned to each treatment group, and then TS at either 5 mg/kg, TS at 50 mg/kg, GV at 2 mg/kg or TS at 5 mg/kg plus GV at 2 mg/kg was administered every other day via IP injection until control mice were moribund. The results for each treatment group were compared to 6 mice receiving vehicle control (DMSO).

**Fig 6:**
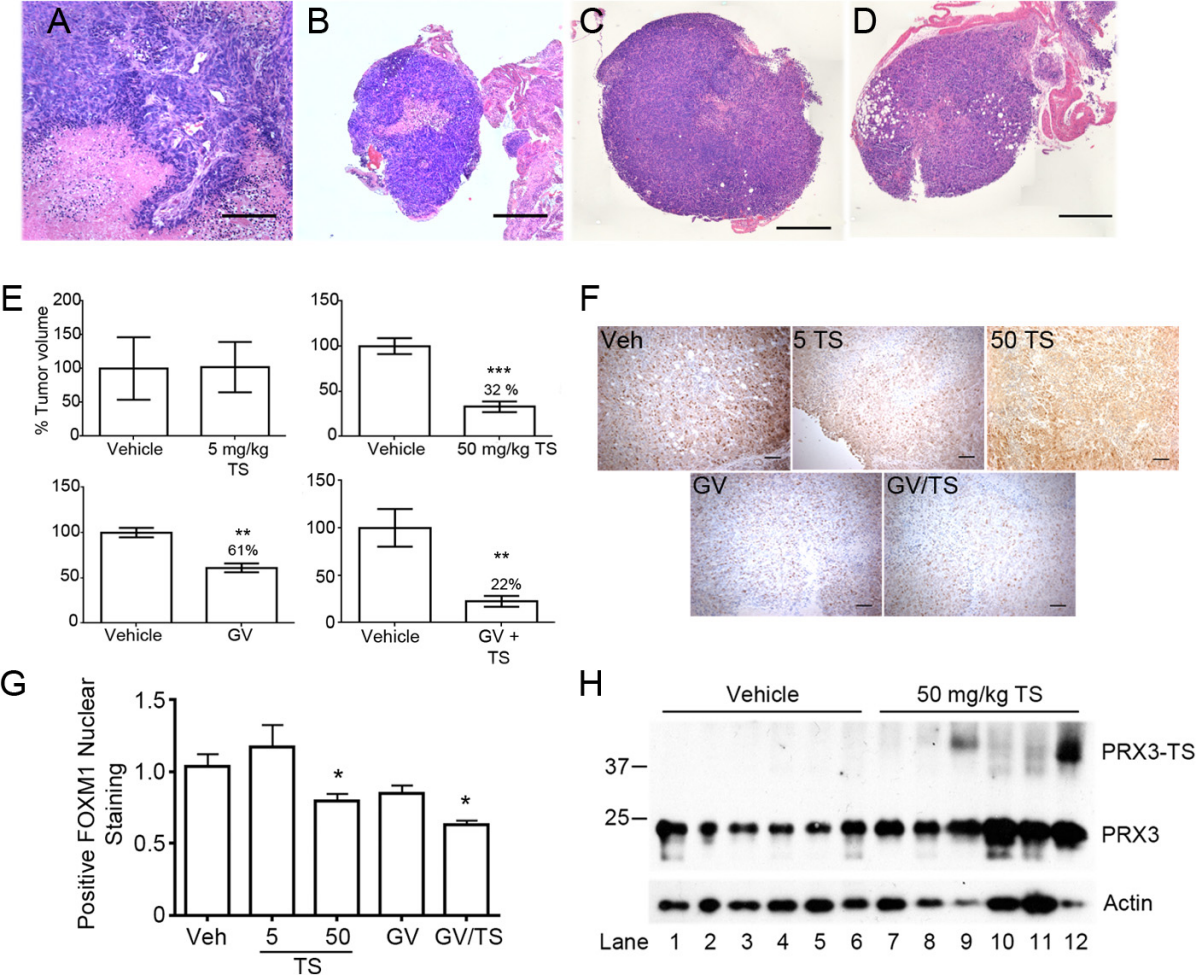
TS and gentian violet reduce tumor volume in a SCID mouse xenograft model of MM. (A) Hematoxylin and eosin (H&E) stained tumor tissue isolated from the peritoneal cavity of a DMSO control Fox Chase SCID mouse 5 weeks after injection of human HM cells. MM tumors were of biphasic morphology with necrotic areas that stained with eosin (pink staining). Tumors often contained stromal tissue of mouse origin and locally invaded the pancreas, liver and omentum. (B-D) Representative H & E stained tumor tissue from mice treated with 50 mg/kg TS, 2 mg/kg GV, or 5 mg/kg TS plus 2 mg/kg GV, respectively, every other day for 21–25 days. Tumor architecture and morphology was similar between treated and untreated animals (scale bar = 0.5 mm). (E) Tumor volumes from animals treated with 5 mg/kg TS, 50 mg/kg TS, 2mg/kg GV, or 2 mg/kg GV plus 5 mg/kg TS are presented as percent of vehicle control (DMSO) (n = 4–6 animals per group, ** p < 0.01, *** p < 0.001). (F) Representative images of FOXM1 immunohistochemistry used for nuclear quantification of FOXM1 from indicated tumor tissue (scale bar = 100 μm). (G) Quantification of FOXM1 positive nuclei from indicated treated tumor sections expressed as relative to vehicle (Veh) (n = 5, * p < 0.05). (H) Immunoblot of PRX3 expression in HM tumor lysates after reducing SDS-PAGE. Error bars represent SEM. See also S5 Fig.

In the IP model MM tumors grew as both small free-floating spheroids and as larger, multi-lobulated solid malignancies that populated mesenteric surfaces and often invaded the surface and interstitium of the pancreas, diaphragm and liver (S5A–S5F Fig). IP tumors also occasionally contained stromal tissue of mouse origin. Tumors were of biphasic MM morphology, and tumors often contained large areas of necrosis that stained positively for eosin (Fig 6A–6D). In contrast to the results with subcutaneous MM tumor model, administration of TS at 5 mg/kg every other day by IP injection had no significant effect on tumor volume in the IP model (Fig 6E). At 50 mg/kg, however, TS showed a significant effect on tumor volume, reducing average tumor volume to ~32% of that observed for vehicle controls (Fig 6E). These results are consistent with findings that administration of 40 mg/kg of TS led to significant decrease in tumor growth in xenograft models of breast, ovarian and hepatocellular carcinoma [48–50]. Treatment with 2 mg/kg GV also resulted in a significant response; reducing tumor volume in treated animals to an average of 61% of controls. The most dramatic response was observed in mice treated with 2 mg/kg GV plus 5 mg/kg TS, a regimen that reduced tumor volume after 21 days to ~ 22% of vehicle control.

Immunohistochemical analysis of nuclear FOXM1 expression with ImageJ in IP tumors from animals treated with 5 mg/kg TS or 2 mg/kg GV did not reveal profound differences in expression, but did reveal diminished expression of nuclear FOXM1 for animals treated with 50 mg/kg TS or the combination of TS and GV (Fig 6F and 6G). We note, however, that diffuse cytoplasmic staining of FOXM1 was evident in the tumors from both the vehicle control and treatment groups. Effects of 50 mg/kg TS on PRX3 expression levels by immunohistochemistry showed variable levels of PRX3 staining, with a tendency for increased staining at the periphery of tumors from both vehicle controls and treatment groups (S5G Fig). Immunoblotting of extracts of tumors from animals treated with 50 mg/kg TS revealed the presence of TS-modified forms of PRX3, which were not present in extracts of control tumors (Fig 6H). Modified species of PRX3 were not detected in extracts of tumors from mice treated with 2 mg/kg GV, or with 5 mg/kg TS with or without GV (not shown). Overall, the morphology and architecture of MM tumors from treated animals were similar to those from control animals, albeit significantly smaller in total volume. Nonetheless, these results clearly demonstrate that administration of GV and TS together was more effective than either agent alone.

## Discussion

Controlled and localized production of cellular hydrogen peroxide is required for growth factor signaling and cell cycle progression, and redox-responsive signaling pathways are known to be involved in cell differentiation, autophagy, migration and survival, all processes relevant to carcinogenesis. A pro-oxidant state is detrimental to normal cells, and therefore oxidant levels are managed by a broad array of small molecules and a repertoire of antioxidant enzymes that include catalase, superoxide dismutase, glutathione peroxidases and peroxiredoxins. Because tumor cells generally produce high levels of oxidants, most often as a consequence of perturbations in energy metabolism [16], they require adaptive responses to survive and benefit from a pro-oxidative state. These responses are not always intuitive. For example, catalase expression and activity is extinguished in lung cancer [51], whereas the cytoplasmic peroxidase PRX1 is highly over-expressed [52]. Given that it is a common feature of many tumor types, oxidant metabolism has emerged as an important therapeutic target, with accentuation of oxidant production that overwhelms antioxidant reserve capacity showing the most clinical promise [11].

The PRX family of thiol peroxidases has gained considerable prominence in the regulation of redox signaling and tumor cell biology. PRXs interact with a wide variety of kinases, growth factor receptors, phosphatases and other regulatory proteins and thereby govern their redox state [53]. Given their role in regulating redox signaling, and the pro-oxidative state of tumor cells, it is not unexpected that the expression of PRXs is up-regulated as an adaptive response in many tumor types [54,55]. We have focused on this adaptive response due to its almost universal existence in tumor cells [16].

The anticancer mechanism of action for TS has been attributed to inhibiting the expression of the oncogenic transcription factor FOXM1 [28]. TS has been proposed to inhibit the proteasome [32], perhaps as a consequence of proteotoxic and oxidative stress [30,33]. TS also has been proposed to bind FOXM1 directly and inhibit its transcriptional activity [31]. We found that the activity of TS in mesothelioma cells is redox-dependent, and that TS modifies the electrophoretic mobility of the mitochondrial peroxidase PRX3. Modification is enhanced by compounds targeted to mitochondria that promote mitochondrial superoxide production [56] or inhibit the expression of TRX2 [34], the primary reductant for PRX3. TS increases the production of hydrogen peroxide in isolated mitochondria respiring on succinate (Fig 1), a response consistent with inactivation of PRX3 peroxidase activity via adduction of the peroxidatic and resolving cysteine residues, Cys108 and Cys229, (Figs 2 and 3) within the PRX3 dimer.

**Fig 7:**
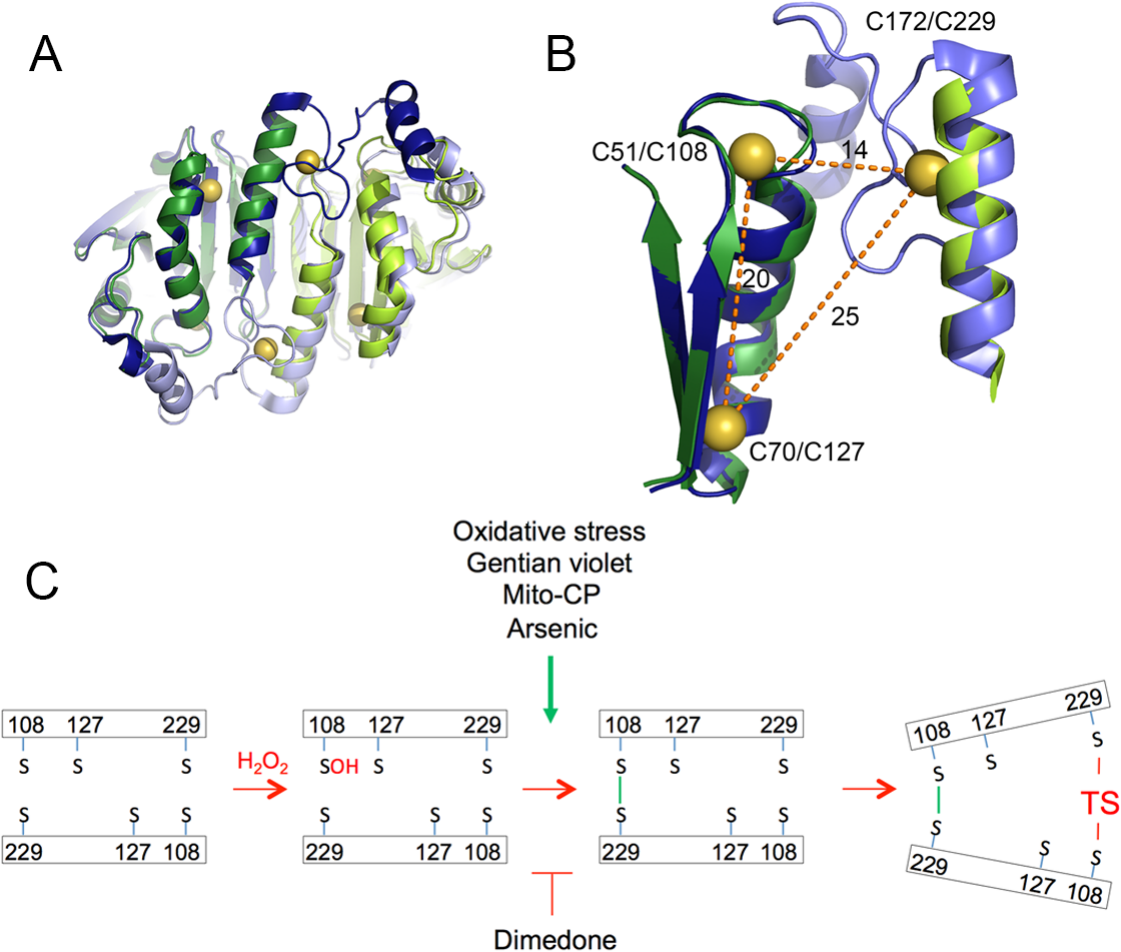
A catalytic intermediate of PRX3 is a molecular target of thiostrepton. (A) Superposition of human Prx2 and bovine Prx3. The monomers of the Prx2 and Prx3 dimer are shown in blue/light blue and green/light green, respectively. The sulfur atoms of the Cys residues are shown as spheres. (B) Proximity of Cys residues. All Cys residues are conserved. The residue numbers indicated are for human Prx2/Prx3. Distances shown are in Angstroms. PDB codes 1QMV and 1ZYE. Note that the C-terminus of Prx3 is disordered and not shown. (C) Model for TS adduction of Prx3. During the PRX3 reaction cycle the formation of a disulfide bond at one catalytic dyad promotes local unfolding. We propose that this conformation change favors adduction of Cys108 and Cys229 in the neighboring catalytic center by TS, leading to an irreducible, crosslinked PRX3 dimer and loss of peroxidase activity. Pro-oxidant compounds such as gentian violet, Mito-CP or arsenic trioxide and oxidative stress increase the level of PRX3 disulfide-bonded dimers and promote adduction by TS. Dimedone attacks sulfenic acid moieties and blocks disulfide formation, thereby blocking modification of PRX3. Similarly, mutant forms of PRX3 lacking the peroxidatic or resolving cysteines are not targets of TS.

Our data suggest a model for the modification of PRX3 by TS that provides a physiological basis for its selective effects on tumor cells (Fig 7). PRXs function as head-to-tail homodimers, with two reaction sites in opposite orientations. Hence, the peroxidatic cysteine in the N-terminus of one monomer forms a disulfide bond with the resolving cysteine in the carboxy terminus of the opposing subunit, and vice versa (Fig 7A and 7B). [57]. Our observations that dimedone (Fig 2) reduces the formation of TS-induced modification of PRX3, whereas GV increases modification, indicate that a specific catalytic intermediate in the PRX3 reaction cycle is the preferred target of TS. Structural studies by others have shown that formation of a disulfide bond between the peroxidatic cysteine and resolving cysteine results in a change from a fully-folded to a locally-unfolded conformation [36]. We propose that this conformational change promotes access of TS to the neighboring catalytic center (Fig 7), thereby positioning dehydroalanine moieties in proximity to the peroxidatic Cys108 and resolving Cys229 residues, resulting in an irreversibly crosslinked PRX3 homodimer. Under any condition investigated to date with purified protein, TS-adduct formation can only be observed under conditions that were designed to promote PRX3 turnover (Fig 2), suggesting that only a small fraction of PRX3 molecules are modified per turnover. Lower rates of PRX3 turnover in primary HMCs and LP9 cells likely account for reduced sensitivity to TS (Fig 4). In contrast, when MM cells, which produce significantly more mitochondrial H_2_O_2_, are treated with high concentrations of TS and GV, virtually the entire cellular pool of PRX3 can be modified by TS. These results suggest the rate of hydrogen peroxide flux dictates the susceptibility of PRX3 to adduction by TS in living cells.

MM cells have increased mitochondrial ROS [34], altered antioxidant enzyme expression and activity [34,58] and reduced mitochondrial reserve capacity (Fig 1), phenotypic features that support tumorigenesis at a cost of redox vulnerability [11]. We propose the increased sensitivity of TS and GV in MM tumor cells compared to LP9 immortalized non-tumorigenic and primary mesothelial cells is due to this inherent difference in basal redox status. MM tumor cell death was apparent at a concentration range of 1–2 μM for either compound, while 6–30 μM was required to kill non-MM cell lines (Fig 4). These values correlated with the formation of modified PRX3 in normal and MM cell lines, and the inability of GV to accentuate formation of PRX3-TS complexes in primary cells may be due to lower levels of the disulfide-bonded dimer intermediate (Fig 4). Hence, to date agents that increase mitochondrial superoxide production and promote catalytic cycling of PRX3, and consequently increase the abundance of disulfide-bonded dimers, have been found to promote PRX3-TS complex formation. As for GV, Mito-Carboxy Proxyl and Mito TEMPOL, both of which increase mitochondrial superoxide production, increase adduction by TS [56]. Interestingly, under normal conditions disulfide-bonded PRX3 dimers appear to be relatively long lived. After cessation of acute oxidative stress, disulfide-bonded PRX3 dimers persist for several hours in mouse lung epithelial cells, and their rate of reduction is dictated by the activity of TR2 [59]. Thus, the presumed catalytic intermediate targeted by TS is both present and persistent in MM tumor cells. In contrast, normal cells that do not constitutively produce high levels of mitochondrial oxidants are less reliant on the TR2-TRX2-PRX3 network, and therefore do not accumulate the PRX3 catalytic intermediate that is the preferred target of TS.

Recent studies with increased temporal specificity support our model for the activity of TS. TS has been shown to increase ROS levels and deplete glutathione levels in malignant melanoma cells prior to the onset of proteasome inhibition, a response also prevented by pre-incubation with NAC [30]. TS is known to target mitochondrial ribosomes [60] and inhibits mitochondrial protein translation [33], showing TS accumulates in mitochondria. An unresolved issue is how TS reaches mitochondria, both in cells in culture and tumor cells *in vivo*, without being inactivated by cellular thiols. Both GSH and NAC interact with TS *in vitro* to some degree (S1 Fig), and this may explain the protection elicited in cell culture by thiol-containing compounds such as NAC. Tumor cells are under increased oxidative stress, tipping the ratio of GSH to GSSG to a more oxidized state, but even under these conditions the cytoplasmic levels of GSH would be expected to inactivate TS. Nonetheless, micromolar concentrations of TS clearly gain access to PRX3 in the mitochondria.

Finally, our data indicate PRX3 is an important and relevant molecular target of TS. As for other cell types, knockdown of PRX3 impedes the proliferation of HM cells (Fig 5). Even accounting for reduced rates of proliferation, shPRX3 HM cells were significantly less sensitive to TS (Fig 5), and expression of catalase in this background restored cell proliferation without restoring sensitivity to TS. Knockdown of PRX3 expression also inhibited the expression FOXM1 mRNA and protein, suggesting a relationship between the regulation of PRX3 and expression of cytoplasmic FOXM1. Given that FOXM1 regulates transcription of the PRDX3 gene [27], we favor a model where PRX3 participates in retrograde signaling to the nucleus that controls a feedback loop in which FOXM1 promotes transcription of its own mRNA [61]. We have recently begun to tease apart a relationship between PRX3, mitochondrial architecture and redox-dependent cell cycle progression. shPRX3 HM cells harbor hyperfused mitochondria and arrest in mitosis due to excessive mitochondrial ROS levels [62]. To better understand how PRX3 functions in balancing ROS levels during the cell cycle, mitochondrial morphology and FOXM1 expression will require further research.

TS when administrated intraperitoneal at 50 mg/kg to mice harboring human MM tumor xenografts significantly decreased nuclear FOXM1 expression and tumor volume compared to vehicle and 5 mg/kg TS treated animals (Fig 6). In agreement with our *in vitro* findings that inhibition of TRX2 with GV potentiates PRX3 adduction (Fig 2) and sensitizes MM cells to TS [34], administrating a combination of TS and GV led to the largest reduction in tumor volume *in vivo* (Fig 6). Targeting multiple nodes in the same tumorigenic pathway has been proposed to be a more effective approach than targeting a single molecule in the pathway [63]. Furthermore, our combinatorial approach of targeting two antioxidant enzymes required to maintain mitochondrial redox homeostasis has broad applicability, as increased ROS and redox vulnerability are phenotypic hallmarks in the majority of cancers [10,11]. Detection of the modified form of PRX3 in xenograft MM tumors from animals treated with 50 mg/kg TS provides evidence that the mechanism of action *in vivo* is similar to that *in vitro*. If so, the modified form of PRX3 may prove to be a useful biomarker for clinically approved agents like arsenic trioxide that promote mitochondrial oxidative stress and enhance the anti-cancer activity of TS. Moreover, determining the levels of mitochondrial oxidants that dictate biological outcomes in response to TS may provide a bioassay for understanding how cancer cells develop a dynamic balance between mitochondrial oxidant production and metabolism to fuel growth.

## Materials and Methods

### Materials

Thiostrepton was purchased from EMB Biochemicals (Billerica, MA). Gentian violet was a kind gift from J. Arbiser (Emory University, Atlanta, GA). Dimedone, glutathione, N-acetyl-L-cysteine, and N-ethyl maleimide were purchased from Sigma (St. Louis, MO). Tris (2-carboxyethyl) phosphine (TCEP) was purchased from Thermo Scientific (Rockford, IL).

### Cell Culture

Human malignant mesothelioma cell lines (HM, H2373) and immortalized but non-tumorigenic mesothelial cells (LP9) were cultured as previously described [56]. Human primary mesothelial cells were isolated from ascites fluid from patients admitted to Interventional Radiology via paracentesis and thoracentesis with no history of smoking or thoracic cancer. Acites fluid was processed for the isolation and cultivation of mesothelial cells as previously described [64]. All protocols for the collection of human specimens were approved by the University of Vermont Institutional Review Board, and specimens were obtained with permission from patients as reflected by signed informed consents.

### Mitochondrial Isolations

Rat heart mitochondria were prepared by homogenization in STE buffer (250 mM sucrose, 5 mM Tris-HCl, 1 mM EGTA, 0.1% fatty acid-free BSA, pH 7.4) using an Ultraturrax blender followed by differential centrifugation as described [65]. Protein concentration was measured by the biuret assay with bovine serum albumin (BSA) as a standard.

### Amplex Red assay

H_2_O_2_ production from isolated mitochondria was measured by horseradish peroxidase oxidation of Amplex Red to fluorescent resorufin [66]. Mitochondria were incubated with stirring at 37°C in standard assay medium (250 mM sucrose, 5 mM HEPES, 1 mM EGTA, 0.01% BSA, pH 7.4 NaOH) containing 50 μM Amplex Red (Molecular Probes) and 4 units/ml horseradish peroxidase. Resorufin was monitored continuously in a fluorimeter (Shimadzu Rf-5301PC) (ex = 560 nm, em = 590 nm). To generate endogenous mitochondrial H_2_O_2_ by reverse electron transport (RET) 10 mM succinate was included in the reaction [37].

### Mitochondrial oxidation state as evaluated by mito-roGFP

MM cells were transfected with mito-roGFP as previously described [56]. The following day media was replaced with CO_2_-independent imaging media containing 134 mM NaCl, 5.4 mM KCl, 1.0 mM MgSO_4_, 1.8 mM CaCl_2_, 20 mM HEPES and 5 mM d-glucose (pH 7.4) and dishes were imaged on a Nikon Ti-E inverted microscope with a 100X 1.49 NA objective in a heated environmental chamber. To determine the oxidation state of the probe, fluorescence images were collected with an Andor iXon X3 EMCCD camera (Andor Technology, Belfast, UK) after excitation with the violet (~400 nm) or teal (~495 nm) outputs from a SpectraXlight engine (Lumencor, Beaverton, OR); emission was collected at 525 nm for both excitation wavelengths. Individual cells were imaged and the ratio of emission from 400 (oxidized) and 495 (reduced) roGFP was measured to determine the relative redox status under indicated experimental conditions.

### Bioenergetic profiles

40,000 cells were plated into individual wells of a XF24 cell culture microplate in complete media excluding cells from 4 wells. The following day cells were washed 1X with XF assay media and 560 μL of assay media was added back to each well. Cells were allowed to equilibrate for 30 mins in a 37°C CO_2_ free incubator before loading into a XF24 extracellular flux analyzer temperature adjusted to 37°C (Seahorse Biosceince, Billerica, MA). Sensor cartridges were equilibrated with XF calibrant for 24 hours before loading with inhibitors. Inhibitor concentrations were titrated to determine optimal drug concentrations to establish bioenergetic profiles (data not shown), final concentrations used were 1 μM oligomycin, 0.75 μM carbonyl cyanide *m*-chlorophenyl hydrazone (CCCP), and 1 μM rotenone and antimycin A. Oxygen and proton concentrations were measured every 8.5 min for 1 hr and 35 min, inhibitors (oligomycin, CCCP, rotenone/antimycin A, respectively) to measure mitochondrial stress were added to the plates through the microinjection ports every 17 min. Oxygen consumption rates (OCR) and extracellular acidification rates (ECAR), as well as reserve capacity (difference between maximal and basal OCR), are shown for 5 replicates and 2 independent experiments. Cells were stained with 4 μg/mL Hoechst 33342 (Life Technologies) following each run to ensure equal cell density (not shown).

### RT-qPCR

Total RNA was extracted using the RNeasy Mini Kit following the manufactures recommended protocol (Qiagen, Hilden, Germany). cDNA was prepared from total RNA using the High Capacity cDNA Reverse Transcription kit following the manufacturers protocol (Life Technologies) and gene expression levels were determined using Assay on Demand TaqMan primers for PRX3 (Hs00428953_g1), FOXM1 (Hs01073586_g1), and HPRT1 (Hs02800695_m1) as a housekeeping control. qPCR was performed on an Applied Biosystems Prism 7900HT Sequence Detection System (Life Technologies) using SDS software (version 2.4).

### Immunofluorescence

HM and HMshPRX3 cells were plated on sterile 18-mm glass coverslips (Fischer Scientific, Pittsburg, PA) and fixed the following day with fresh 3.4% paraformaldehyde and permeabilized with 0.3% Triton X 100. Coverslips were blocked with 3% bovine serum albumin (BSA) in PBS for 1 hr at room temperature (RT). Coverslips were incubated with primary antibodies anti-FOXM1 K-19 and anti-Cox IV (Abcam, Cambridge, UK) diluted 1:200 in 1.5% BSA for 1 hr at RT. Coverslips were washed 5X 5 min with PBS and incubated with secondary goat anti-rabbit 594 (Invitrogen, 1:400) and donkey anti-mouse 488 (Invitrogen, 1:400) antibodies for 1 hr at RT. Coverslips were washed 5X 5 min with PBS, with the final wash containing DAPI (Invitrogen, 1:4000), and mounted on glass slides with Aqua-Poly/Mount (Polysciences, Inc, Warrington, PA). Images were collected on a Nikon Ti-E inverted microscope with a 60x oil immersion objective. Exposure times were adjusted based on secondary antibody controls.

### Cell growth assays

Cells were plated into 96 well plates at a density of 1000 cells/well. The following day cells were stained with 4 μg/mL Hoechst 33342 (Life Technologies) for 10 min, washed with PBS and incubated with fresh complete media. Hoechst 33342 fluorescence was quantified using a Synergy HT plate reader (BioTek, Winooski, VT) ex340/em460. This procedure was repeated for 4 consecutive days.

### Crystal violet assay for total cell mass

Cells were plated in 96-well plates at a density of 1,500 cells per well. The next day, cells were treated as indicated in complete medium. After 24 hr cells were washed with PBS, fixed in 3.7% paraformaldehyde and stained for 30 min with 0.1% crystal violet in water. To quantify crystal violet staining, plates were washed with H_2_O, dried, and the dye was dissolved in 100% methanol, absorbance was read at 540 nm. The Relative Potency (REP) of TS and/or GV was determined using Gen5 software (BioTek Instruments, Winooski, VT) using primary mesothelial cells as reference cell line. REP measurements using Gen5 software are based on a constrained model whereby the software “constrains” the reference and treatment curves so that parameters for non-linear functions can be calculated together. The REP describes the difference between the control and treatment curves to compare two (or more) response curves. The EC_50_ for TS and GV was calculated for each cell line by calculating the concentration of TS needed to reduce cell mass to 50% of untreated.

### Immunoblotting

Tumor tissue lysates were prepared in modified RIPA (mRIPA) buffer. Tissue was homogenized in Lysing Matrix A Tubes (MP Biomedicals Inc. Santa Ana, CA) by shaking for 30 sec at 6.5 m/s on a FastPrep 24 benchtop homogenizer (MP Biomedicals Inc.) and cleared by centrifugation. Cell lysates were prepared by scraping on ice in NP-40 lysis buffer [67] and cleared by centrifugation. 10 μg of cell lysate was resolved by SDS–PAGE and transferred to PVDF membranes. Membranes were prepared for antibody addition following standard protocols and incubated at 4°C overnight with the indicated primary antibodies: PRX3 (1:2000, Ab Frontier, Seoul, Korea), FOXM1 K-19 (1:500, Santa Cruz Biotechnology, Dallas, TX) and actin (1:5000, Millipore, Billerica, MA). Blots were incubated with horseradish peroxidase conjugated secondary antibodies (1:2500) for 30 min at RT and protein bands were visualized with the Western Lightning chemiluminescent detection system (Perkin Elmer, Waltham, MA).

### Immunoprecipitation

Cell lysates from cells treated with DMSO or thiostrepton and gentian violet were prepared in immunoprecipitation (IP) lysis buffer 500 mM Tris-Cl pH 7.5, 150 mM NaCl, 1% NP-40, 0.2% SDS, 0.2% CHAPS, 20 mM N-ethyl maleimide and protease inhibitor cocktail (Roche) and 0.5 mgs of total protein was incubated with 1 μg anti-peroxiredoxin 3 antibody for 1 hr at 4°C on an orbital rocker. 50 μL of equilibrated protein G magnetic Dynabeads (Life Technologies) were added to each tube and rocked for 1 hour at 4°C. Protein/bead complexes were immobilized on a magnetic support and washed 5X with IP buffer, mixing by vortexing between each wash. Proteins were removed from Dynabeads with the addition of 5X sample buffer and heated at 95°C for 10 minutes before separation by SDS-PAGE. 2 μL of a 25 μL total volume was used for western blot to detect complex formation while the remaining volume was used for Coomassie Blue staining and MS analysis. Gel bands were digested by “in gel” cleavage at 37°C with 12.5 ng/mL sequence grade trypsin in buffer consisting of 20 mM Tris-HCl, pH 8.0, and 5 mM CaCl_2_. Peptides were extracted from the gel with a 4% ARISTAR-grade formic acid, 60% acetonitrile solution.

### LC-MS/MS analysis of cellular samples

Samples were prepared as described above and analyzed by LC-MS/MS on a linear ion trap LTQ-Orbitrap XL Mass Spectrometer (Thermo Fisher Scientific, MA). 6 μL of the material was loaded onto a 100 μm x 120 mm capillary column packed with MAGIC C18 (5 μm particle size, 20 nm pore size, Michrom Bioresources, CA) at a flow rate of 500 nL/min. Peptides were separated by a gradient of 5–35% CH_3_CN/ 0.1% formic acid over 100 minutes, 40–100% CH_3_CN/0.1% formic acid in 5 minutes, and 100% CH_3_CN for 10 minutes. Product ion spectra were searched using the SEQUEST search engine on Proteome Discoverer 1.4 (Thermo Fisher Scientific, MA) against a curated Human database with sequences in forward and reverse orientations. The database was indexed to allow for full trypsin enzymatic activity, two missed cleavages, and peptides between the MW of 350–5000. Search parameters set the mass tolerance at 20 ppm for precursor ions and 0.8 Da for fragment ions. Cross-correlation (Xcorr) significance filters were applied to limit the false positive rates to less than 1% for each sample. Other filters applied were a minimum peptide cutoff of 2 as well as DeltaCN >0.1.

### Mass spectrometry analysis of GSH/NAC thiostrepton adducts

Thiostrepton was reacted glutathione (GSH) or N-acetyl-L-cysteine (NAC) for 1 hr at room temperature in 60% acetonitrile/methanol solution. The reaction mixtures were analyzed by electrospray ionization mass spectrometry on the LTQ mass spectrometer (Thermo Fisher Scientific) in the positive mode. Analyses were performed at a flow rate of 50 μL/min by introducing samples into the LC flow (47 μL/min) using a syringe pump (3 μL/min) with a T-connection. Operating parameters were as follows: spray voltage at 5.0 kV, sheath gas at 8 units, and capillary temperature at 275°C. Full scan mass spectra (*m/z* 100–2000) were acquired with unit resolution with the “Acquire Data Dialog Box”. The results were analyzed with XCalibur program (Thermo). The experimental masses of the analytes were obtained by averaging 50 scans.

### *In vitro* TR-TRX-PRX3 system

The human *PRDX3* gene (residues 62–256) was codon optimized for expression in *Escherichia coli* by GenScript and subcloned into the pET15b vector. The resultant protein (residues 62–256) contained a non-cleavable, N-terminal His-tag. The Cys to Ser variants (C108S, C127S, and C229S) were generated using the QuickChange protocol and the appropriate primers (Stratagene). The proteins were expressed in C41 (DE3) cells and purified using nickel-NTA (Qiagen), Q-Sepharose FF and Superdex 200 columns (both GE Healthcare). The final storage buffer was 25 mM Hepes pH 7.5, 100 mM NaCl. A dimeric Prx3 variant was created by introducing two charged residues into the dimer-dimer interface (S139E/A142E), as was previously done with human Prx1 [68]. The His-tag of the S139E/A142E variant (EE Mut) was removed by digestion with biotinylated thrombin (Novagen). Comparable amounts of thiostrepton adducts were observed in control reactions with either non-tagged [69] or tagged wild-type Prx3. *E*. *coli* thioredoxin reductase (TR) and *E*. *coli* thioredoxin 2 (Trx2, the *trxC* gene product) were expressed and purified as previously described [70].

The *in vitro* reaction contained recombinant 100 μM PRX3, 5 μM *E*. *coli* TRX2, 0.5 μM *E*. *coli* TR, and a NADPH regenerating system composed of 3.2 mM glucose 6-phosphate, 3.2 U/ml glucose 6-phosphate dehydrogenase and 0.4 mM NADPH. Samples were incubated for 16–18 hours at 37°C with either 0.2 mM TS or an equivalent volume of DMSO. Assay components were pulsed with successive additions of 100 μM H_2_O_2_ to induce turnover of PRX3. Reactions were stopped by the addition of 5X sample buffer containing 100 mM DTT, heated to 100°C for 10 minutes, and proteins were separated by SDS-PAGE with sodium bisulfate present in the running buffer and stained for total protein using GelCode Blue (Life Technologies).

### Mass spectrometry of rPRX3 variants

For analysis of the intact rPrx3-TS adduct, the EE Mut of Prx3 was cycled with and without TS as described for the *in vitro* assay and then treated with 33 mM DTT for 30 minutes at room temperature. Samples were concentrated and re-diluted 9 times using a 0.5 ml Amicon Ultra centrifugation filter (10,000 MWC) to exchange into 25 mM ammonium bicarbonate for MS analysis. ESI-TOF MS analyses were performed on an Agilent 6120 MSD-TOF system (Agilent Technologies, Inc., Santa Clara, CA, USA) operating in positive ion mode with the following settings: capillary voltage 3500 V, nebulizer gas pressure 30 psig, drying gas flow 5 L min^−1^, fragmentor voltage 175 V, skimmer voltage 65 V, gas temperature 325°C. Samples were introduced via direct infusion at a flow rate of 20 μL min^−1^ using a syringe pump (KD Scientific, Holliston, MA, USA). For peptide analysis, the samples utilized for ESI-MS analysis (~30 μg protein) were digested with 1.5 μg Pierce MS-grade trypsin overnight at 37°C. Peptides were analyzed on a Bruker Autoflex MALDI-TOF mass spectrometer in positive ion and linear acquisition mode using sinapic acid as the matrix.

### Generation of shPRX3, shPRX3/pZeo, shPRX3/Catalase, shPRX3/mito-catalase stable cell lines and si-RNA to PRX3

On-Targetplus human PRDX3 siRNA and scramble control RNA (Thermo Scientific, Waltham, MA) transfection complexes were prepared in Optimem and Lipofectamine 2000 (Life Technologies). Images of siPRX3 cells were acquired using a light microscope equipped with a CCD camera 48 and 72 hr after transfection. PRX3 and pLKO.1 lentiviral shRNAs (Sigma) were packaged following the manufacturers protocol (Sigma). To establish stable shPRX3 and pLKO.1 (shCtrl) cell lines, 1.25 X 10^5^ HM or H2373 cells were plated into 35 mm tissue culture dishes and allowed to adhere overnight. The following day 150 μL of medium containing shPRX3 lentiviral particles or shCtrl particles was added to cells for 18 hr. Virus particles were removed by washing and cells were incubated with complete media. After 2 days culture media was replaced with complete media containing 2μg/mL puromycin and subsequently changed every 2 days until control non-transduced cells were completely non-viable. shPRX3/Catalase (CAT) and shPRX3/mito-Catalase (mCAT) stable cell lines were generated by transfection of shPRX3 cells with expression plasmids for catalase, or mito-Catalase as described above; the empty pZeo vector was used as control. After 2 days culture media was replaced with complete media containing 2 μg/mL Zeocin (Invitrogen) and subsequently changed every 2 days until control non-transfected cells were non-viable

### Xenograft model of human malignant mesothelioma

Male Fox Chase (CB17/lcr-*Prkdc* ^*scid*^/lcrlcoCr background) severe combined immunodeficient (SCID) mice between 6–8 weeks old (Charles River Laboratories, Wilmington, MA) were injected with 2 to 5 x 10^6^ HM cells intraperitoneal (IP) into the lower left quadrant or subcutaneously (SQ) at 2 caudal and 2 rostral sites (SQ tumors were allowed to form for 2 weeks and subsequently treated with 5 mg/kg TS intraperitoneally). After 2 weeks animals from the IP model were treated with 5 mg/kg TS, 50 mg/kg TS, 2 mg/kg GV, 2 mg/kg GV plus 5 mg/kg TS, or 10% DMSO in PBS (Vehicle) every other day for 3–4 weeks (6 animals per group). After 3–4 weeks, mice were euthanized by IP administration of sodium pentobarbital. Free-floating spheroidal and mesenteric tumors were recovered by surgical resection, and tumor volume was measured using the formula length x width x height x π/6. Tumor tissue was fixed in 4% paraformaldehyde in PBS for processing and immunohistochemical assays. All protocols used in animal experiments were approved by the University of Vermont College of Medicine Institutional Animal Care and Use Committee (IACUC).

### Immunohistochemistry (IHC)

Tumor and tissue sections were deparaffinized in xylene (3 x 15 minutes) and subsequently rehydrated in a graded ethanol series (100% to 50% ethanol). Slides were then subjected to antigen retrieval using a 1X DAKO solution (Dako, Glostrup, Denmark) at 96°C according to the manufacturer’s specifications. IHC was performed using antibodies to FOXM1 C-20 (Santa Cruz Biotechnology), and PRX3 (Ab Frontier) according to the manufacturers’ specifications; incubations with these antibodies were performed for half an hour. 3’3-diaminobenzidine (DAB) was then applied to each slide, rinsed off, and counterstained with hematoxylin prior to fixing with Aqua-Poly/Mount (Polysciences Inc., Warrington, PA), and coverslipped. Tumor sections were imaged using an Olympus BX50 upright light microscope (Olympus America, Lake Success, NY) with an attached Optronics MagnaFire digital camera (Optical Analysis, Nashua, NH).

### FOXM1 nuclear staining quantification

FOXM1 IHC sections were imaged as described above using a 10X objective. RGB images were converted to 8-bit images and equally thresholded to isolate dark nuclei. Nuclei were then counted using the particle analysis plugin of ImageJ (ImageJ, NIH) restraining the particle size to 0.0005–0.005 inches^2^ (empirically determined based on surveying the relative sizes of various nuclei). Data are expressed as relative to vehicle controls.

### Statistical analysis

Data are presented as +/- SEM or +/- SD where indicated. Statistical significance was determined using 1-way ANOVA with a Tukey’s post-hoc test or the students *t* test comparing control to experimental conditions for p<0.05.

## Supporting Information

S1 Fig immunoprecipitation of PRX3-TS complex from HM cells and adduction of TS by thiols. (A) Whole cell lysate (WCL) from HM cells treated with 5 μM TS and 1 μM gentian violet (T/G) were resolved by reducing and denaturing PAGE and immunoblotted for PRX3 (Lanes 1 and 2). PRX3 was immunoprecipitated from 100 μg of total protein from control cell lysates (0) and TS/GV lysates and resolved by reducing and denaturing PAGE (lanes 3 and 4) (B) Bands corresponding to PRX3 monomers (~23 kD) and dimers (~40 kD) were recovered from the gel shown in panel C and digested with trypsin. Peptides corresponding to PRX3 were identified by LC-MS/MS. (C) MS spectrum of thiostrepton (TS, 1664.61 Da). (D) Thiostrepton was incubated with glutathione (GSH, left) or N-acetyl-L-cysteine (NAC, right) in reaction buffer and analyzed by LC–MS.
(TIF)

S2 Fig Bioenergetic profiles for LP9 and HM cells treated with thiostrepton. (A) Oxygen consumption rate (OCR) for LP9 cells treated with or without 5 μM thiostrepton (TS) for 6 hrs. (B) OCR for HM cells treated with or without 5 μM TS for 6 hrs. (C) Extracellular acidification rate (ECAR) for LP9 and HM cells treated with or without TS for 6 hrs. (D) Basal ECAR for LP9 and HM cells with or without TS. Error bars represent SEM.
(TIF)

S3 Fig shPRX3 cells proliferate slower and have reduced FOXM1 expression compared to WT controls. (A) Nuclear staining was used to determine cell number in H2373 cells and shPRX3 H2373 cells (H2shPRX3) over 4 days (n = 4). (B) PRX3 transcript levels in H2373 cells and H2shPRX3 cells (n = 3 * p < 0.05). (C) Nuclear staining was used to determine cell number in HM cells transfected with scramble or FOXM1 siRNA (n = 4, ***p < 0.001). Error bars represent SEM. (D) FOXM1 transcript levels in H2373 cells and H2shPRX3 cells as determined by qRT-PCR (n = 3, * p < 0.05). E) WT and HMshPRX3 cells were fixed and immunostained for FOXM1 and Cox IV (to visualize mitochondrial structures); nuclei were counterstained with DAPI (scale bar = 10 μm). (F) Regions of interest were drawn around the nucleus (Nuc, white circle) and mitochondrial compartment (Cyto/Mito, blue half circle). Mean fluorescence intensity (MFI) is plotted in (G) for representative mitochondrial and nuclear compartments of indicated cell lines (n = 10 cells). Error bars represent SEM.
(TIF)

S4 Fig TS inhibits tumor progression in a subcutaneous SCID mouse xenograft model of MM. A) Fox Chase SCID mice were injected subcutaneously with HM cells as described in Materials and Methods. After tumors became palpable (about 2 weeks) mice were injected IP with 5 mg/kg TS dissolved in 10% dimethylacetamide (10% DMA) or vehicle control every other day for the indicated number of days. Just prior to each TS injection tumor volume was estimated using calipers. At sacrifice, tumors were dissected and tumor volumes were measured; tumor volume in TS treated animals was significantly different from that of controls (n = 6 mice per group, results shown are representative of 2 independent experiments, ***p < 0.001, *** p < 0.01, * p < 0.05). Analysis of lung and liver specimens revealed no evidence of cytotoxicity due to TS treatment. B) Paraffin-embedded tumor sections were processed for immunohistochemical detection of FOXM1 by IHC (scale bar = 50 μm). C) Nuclear FOXM1 expression was quantified by counting the number of cells with positive nuclear staining in 5 quadrants per section (n = 5, ** p < 0.01). Error bars represent SEM.
(TIF)

S5 Fig Expression of FOXM1 in mouse intraperitoneal MM xenografts. A) Free-floating tumor spheroids measured 3–5 mm in diameter and often contained necrotic areas (scale bar = 0.5 mm). B) Tumor spheroids were typically encapsulated by several layers of FOXM1-positive cells. C and D) FOXM1-positive tumor cells often displayed clear areas between cells, a histological feature of MM due to the presence of microvilli. FOXM1-positive tumor tissue was commonly interspersed with stroma characterized by fibroblastic cells, presumably of mouse origin. E and F) Mesenteric tumors often showed evidence of invasion into abdominal organs such as liver and pancreas (scale bar = 50 μm). G) PRX3 immunohistochemistry staining in vehicle and 50 mg/kg TS tumor sections (scale bar top panels = 0.5 mm, bottom sections = 100 μm).
(TIF)

S1 Table Cysteine and Cysteine-thiostrepton containing peptides as determined by Mass spectrometry. n/o = not observed.
(PDF)

## References

[1] Fried L, Arbiser JL (2008) The reactive oxygen-driven tumor: relevance to melanoma. Pigment Cell Melanoma Res 21: 117–122. 10.1111/j.1755-148X.2008.00451.x 18384505

[2] Cheng CW, Kuo CY, Fan CC, Fang WC, Jiang SS, Lo YK, et al (2013) Overexpression of Lon contributes to survival and aggressive phenotype of cancer cells through mitochondrial complex I-mediated generation of reactive oxygen species. Cell Death Dis 4: e681 10.1038/cddis.2013.204 23788038 PMC3702277

[3] Gupta SC, Hevia D, Patchva S, Park B, Koh W, Aggarwal BB (2012) Upsides and downsides of reactive oxygen species for cancer: the roles of reactive oxygen species in tumorigenesis, prevention, and therapy. Antioxid Redox Signal 16: 1295–1322. 10.1089/ars.2011.4414 22117137 PMC3324815

[4] Jones DP (2010) Redox sensing: orthogonal control in cell cycle and apoptosis signalling. J Intern Med 268: 432–448. 10.1111/j.1365-2796.2010.02268.x 20964735 PMC2963474

[5] Finkel T (2011) Signal transduction by reactive oxygen species. J Cell Biol 194: 7–15. 10.1083/jcb.201102095 21746850 PMC3135394

[6] Burhans WC, Heintz NH (2009) The cell cycle is a redox cycle: linking phase-specific targets to cell fate. Free Radic Biol Med 47: 1282–1293. 10.1016/j.freeradbiomed.2009.05.026 19486941

[7] Sena LA, Chandel NS (2012) Physiological roles of mitochondrial reactive oxygen species. Mol Cell 48: 158–167. 10.1016/j.molcel.2012.09.025 23102266 PMC3484374

[8] Lee AC, Fenster BE, Ito H, Takeda K, Bae NS, Hirai T, et al (1999) Ras proteins induce senescence by altering the intracellular levels of reactive oxygen species. J Biol Chem 274: 7936–7940. 10075689 10.1074/jbc.274.12.7936

[9] Cairns RA, Harris IS, Mak TW (2011) Regulation of cancer cell metabolism. Nat Rev Cancer 11: 85–95. 10.1038/nrc2981 21258394

[10] Weinberg F, Hamanaka R, Wheaton WW, Weinberg S, Joseph J, Lopez M, et al (2010) Mitochondrial metabolism and ROS generation are essential for Kras-mediated tumorigenicity. Proc Natl Acad Sci U S A 107: 8788–8793. 10.1073/pnas.1003428107 20421486 PMC2889315

[11] Wondrak GT (2009) Redox-directed cancer therapeutics: molecular mechanisms and opportunities. Antioxid Redox Signal 11: 3013–3069. 10.1089/ARS.2009.2541 19496700 PMC2824519

[12] Warburg O (1956) On the origin of cancer cells. Science 123: 309–314. 13298683 10.1126/science.123.3191.309

[13] Wallace DC (2012) Mitochondria and cancer. Nat Rev Cancer 12: 685–698. 10.1038/nrc3365 23001348 PMC4371788

[14] Weinhouse S (1956) On respiratory impairment in cancer cells. Science 124: 267–269. 13351638 10.1126/science.124.3215.267

[15] Weinhouse S, Wenner CE (1956) Metabolism of neoplastic tissue. IX. An isotope tracer study of glucose catabolism pathways in normal and neoplastic tissues. J Biol Chem 222: 399–414. 13367012

[16] Gorrini C, Harris IS, Mak TW (2013) Modulation of oxidative stress as an anticancer strategy. Nat Rev Drug Discov 12: 931–947. 10.1038/nrd4002 24287781

[17] Dranka BP, Hill BG, Darley-Usmar VM (2010) Mitochondrial reserve capacity in endothelial cells: The impact of nitric oxide and reactive oxygen species. Free Radic Biol Med 48: 905–914. 10.1016/j.freeradbiomed.2010.01.015 20093177 PMC2860730

[18] Dranka BP, Benavides GA, Diers AR, Giordano S, Zelickson BR, Reily C, et al (2011) Assessing bioenergetic function in response to oxidative stress by metabolic profiling. Free Radic Biol Med 51: 1621–1635. 10.1016/j.freeradbiomed.2011.08.005 21872656 PMC3548422

[19] Diers AR, Higdon AN, Ricart KC, Johnson MS, Agarwal A, Kalyanaraman B, et al (2010) Mitochondrial targeting of the electrophilic lipid 15-deoxy-Delta12,14-prostaglandin J2 increases apoptotic efficacy via redox cell signalling mechanisms. Biochem J 426: 31–41. 10.1042/BJ20091293 19916962 PMC3079364

[20] Cox AG, Winterbourn CC, Hampton MB (2010) Mitochondrial peroxiredoxin involvement in antioxidant defence and redox signalling. Biochem J 425: 313–325. 10.1042/BJ20091541 20025614

[21] Watabe S, Hiroi T, Yamamoto Y, Fujioka Y, Hasegawa H, Yago N, et al (1997) SP-22 is a thioredoxin-dependent peroxide reductase in mitochondria. Eur J Biochem 249: 52–60. 9363753 10.1111/j.1432-1033.1997.t01-1-00052.x

[22] Wood ZA, Schroder E, Robin Harris J, Poole LB (2003) Structure, mechanism and regulation of peroxiredoxins. Trends Biochem Sci 28: 32–40. 12517450 10.1016/s0968-0004(02)00003-8

[23] Chang TS, Cho CS, Park S, Yu S, Kang SW, Rhee SG (2004) Peroxiredoxin III, a mitochondrion-specific peroxidase, regulates apoptotic signaling by mitochondria. J Biol Chem 279: 41975–41984. 15280382 10.1074/jbc.M407707200

[24] Chua PJ, Lee EH, Yu Y, Yip GW, Tan PH, Bay BH (2010) Silencing the Peroxiredoxin III gene inhibits cell proliferation in breast cancer. Int J Oncol 36: 359–364. 20043069

[25] Song IS, Kim HK, Jeong SH, Lee SR, Kim N, Rhee BD, et al (2011) Mitochondrial Peroxiredoxin III is a Potential Target for Cancer Therapy. Int J Mol Sci 12: 7163–7185. 10.3390/ijms12107163 22072940 PMC3211031

[26] Ummanni R, Barreto F, Venz S, Scharf C, Barett C, Mannsperger HA, et al (2012) Peroxiredoxins 3 and 4 are overexpressed in prostate cancer tissue and affect the proliferation of prostate cancer cells in vitro. J Proteome Res 11: 2452–2466. 10.1021/pr201172n 22424448

[27] Park HJ, Carr JR, Wang Z, Nogueira V, Hay N, Tyner AL, et al (2009) FoxM1, a critical regulator of oxidative stress during oncogenesis. EMBO J 28: 2908–2918. 10.1038/emboj.2009.239 19696738 PMC2760115

[28] Kwok JM, Myatt SS, Marson CM, Coombes RC, Constantinidou D, Lam EW (2008) Thiostrepton selectively targets breast cancer cells through inhibition of forkhead box M1 expression. Mol Cancer Ther 7: 2022–2032. 10.1158/1535-7163.MCT-08-0188 18645012

[29] Bhat UG, Halasi M, Gartel AL (2009) FoxM1 Is a General Target for Proteasome Inhibitors. PLoS ONE 4: e6593 10.1371/journal.pone.0006593 19672316 PMC2721658

[30] Qiao S, Lamore SD, Cabello CM, Lesson JL, Munoz-Rodriguez JL, Wondrak GT (2012) Thiostrepton is an inducer of oxidative and proteotoxic stress that impairs viability of human melanoma cells but not primary melanocytes. Biochem Pharmacol 83: 1229–1240. 10.1016/j.bcp.2012.01.027 22321511 PMC3299892

[31] Hegde NS, Sanders DA, Rodriguez R, Balasubramanian S (2011) The transcription factor FOXM1 is a cellular target of the natural product thiostrepton. Nat Chem 3: 725–731. 10.1038/nchem.1114 21860463

[32] Gartel AL (2010) A new target for proteasome inhibitors: FoxM1. Expert Opin Investig Drugs 19: 235–242. 10.1517/13543780903563364 20074015 PMC3532816

[33] Bowling BD, Doudican N, Manga P, Orlow SJ (2008) Inhibition of mitochondrial protein translation sensitizes melanoma cells to arsenic trioxide cytotoxicity via a reactive oxygen species dependent mechanism. Cancer Chemother Pharmacol 63: 37–43. 10.1007/s00280-008-0705-y 18297286 PMC2749296

[34] Newick K, Cunniff B, Preston K, Held P, Arbiser J, Pass H, et al (2012) Peroxiredoxin 3 is a redox-dependent target of thiostrepton in malignant mesothelioma cells. PLoS One 7: e39404 10.1371/journal.pone.0039404 22761781 PMC3382597

[35] Zhang X, Zheng Y, Fried LE, Du Y, Montano SJ, Sohn A, et al (2011) Disruption of the mitochondrial thioredoxin system as a cell death mechanism of cationic triphenylmethanes. Free Radic Biol Med 50: 811–820. 10.1016/j.freeradbiomed.2010.12.036 21215310 PMC3047390

[36] Perkins A, Nelson KJ, Williams JR, Parsonage D, Poole LB, Karplus PA (2013) The sensitive balance between the fully folded and locally unfolded conformations of a model peroxiredoxin. Biochemistry 52: 8708–8721. 10.1021/bi4011573 24175952 PMC3932808

[37] Hurd TR, Prime TA, Harbour ME, Lilley KS, Murphy MP (2007) Detection of Reactive Oxygen Species-sensitive Thiol Proteins by Redox Difference Gel Electrophoresis. J Biol Chem 282: 22040–22051. 17525152 10.1074/jbc.M703591200

[38] Friedrich T, van Heek P, Leif H, Ohnishi T, Forche E, Kunze B, et al (1994) Two binding sites of inhibitors in NADH: ubiquinone oxidoreductase (complex I). Relationship of one site with the ubiquinone-binding site of bacterial glucose:ubiquinone oxidoreductase. Eur J Biochem 219: 691–698. 8307034 10.1111/j.1432-1033.1994.tb19985.x

[39] Hill BG, Benavides GA, Lancaster JR Jr., Ballinger S, Dell'Italia L, Jianhua Z, et al (2012) Integration of cellular bioenergetics with mitochondrial quality control and autophagy. Biol Chem 393: 1485–1512. 23092819 10.1515/hsz-2012-0198 PMC3594552

[40] Cox AG, Peskin AV, Paton LN, Winterbourn CC, Hampton MB (2009) Redox potential and peroxide reactivity of human peroxiredoxin 3. Biochemistry 48: 6495–6501. 10.1021/bi900558g 19462976

[41] Cox AG, Pearson AG, Pullar JM, Jonsson TJ, Lowther WT, Winterbourn CC, et al (2009) Mitochondrial peroxiredoxin 3 is more resilient to hyperoxidation than cytoplasmic peroxiredoxins. Biochem J 421: 51–58. 10.1042/BJ20090242 19356151 PMC3745641

[42] Allison WS (1976) Formation and reactions of sulfenic acids in proteins. Accounts Chem Res 9: 293–299.

[43] Bausch SL, Poliakova E, Draper DE (2005) Interactions of the N-terminal domain of ribosomal protein L11 with thiostrepton and rRNA. J Biol Chem 280: 29956–29963. 15972821 10.1074/jbc.M504182200

[44] Friedman M, Finley JW, Yeh LS (1977) Reactions of proteins with dehydroalanines. Adv Exp Med Biol 86B: 213–224. 20747 10.1007/978-1-4757-9113-6_15

[45] Younis IR, Elliott M, Peer CJ, Cooper AJ, Pinto JT, Konat GW, et al (2008) Dehydroalanine analog of glutathione: an electrophilic busulfan metabolite that binds to human glutathione S-transferase A1-1. J Pharmacol Exp Ther 327: 770–776. 10.1124/jpet.108.142208 18791061 PMC2678891

[46] Chiu ML, Folcher M, Griffin P, Holt T, Klatt T, Thompson CJ (1996) Characterization of the Covalent Binding of Thiostrepton to a Thiostrepton-Induced Protein from Streptomyces lividans Biochemistry 35: 2332–2341. 8652574 10.1021/bi952073e

[47] Kinnula VL, Lehtonen S, Sormunen R, Kaarteenaho-Wiik R, Kang SW, Rhee SG, et al (2002) Overexpression of peroxiredoxins I, II, III, V, and VI in malignant mesothelioma. J Pathol 196: 316–323. 11857495 10.1002/path.1042

[48] Wang M, Gartel AL (2011) Micelle-encapsulated thiostrepton as an effective nanomedicine for inhibiting tumor growth and for suppressing FOXM1 in human xenografts. Mol Cancer Ther 10: 2287–2297. 10.1158/1535-7163.MCT-11-0536 21903609 PMC3237785

[49] Wang M, Halasi M, Kabirov K, Banerjee A, Landolfi J, Lyubimov AV, et al (2012) Combination treatment with bortezomib and thiostrepton is effective against tumor formation in mouse models of DEN/PB-induced liver carcinogenesis. Cell Cycle 11: 3370–3372. 10.4161/cc.21290 22894930 PMC3466545

[50] Chan DW, Hui WW, Cai PC, Liu MX, Yung MM, Mak CS, et al (2012) Targeting GRB7/ERK/FOXM1 signaling pathway impairs aggressiveness of ovarian cancer cells. PLoS One 7: e52578 10.1371/journal.pone.0052578 23285101 PMC3527599

[51] Chung-man Ho J, Zheng S, Comhair SA, Farver C, Erzurum SC (2001) Differential expression of manganese superoxide dismutase and catalase in lung cancer. Cancer Res 61: 8578–8585. 11731445

[52] Chang JW, Jeon HB, Lee JH, Yoo JS, Chun JS, Kim JH, et al (2001) Augmented expression of peroxiredoxin I in lung cancer. Biochem Biophys Res Commun 289: 507–512. 11716502 10.1006/bbrc.2001.5989

[53] Rhee SG, Woo HA (2011) Multiple functions of peroxiredoxins: peroxidases, sensors and regulators of the intracellular messenger H(2)O(2), and protein chaperones. Antioxid Redox Signal 15: 781–794. 10.1089/ars.2010.3393 20919930

[54] Karihtala P, Mantyniemi A, Kang SW, Kinnula VL, Soini Y (2003) Peroxiredoxins in breast carcinoma. Clin Cancer Res 9: 3418–3424. 12960131

[55] Basu A, Banerjee H, Rojas H, Martinez SR, Roy S, Jia Z, et al (2011) Differential expression of peroxiredoxins in prostate cancer: consistent upregulation of PRDX3 and PRDX4. Prostate 71: 755–765. 10.1002/pros.21292 21031435 PMC3107902

[56] Cunniff B, Benson K, Stumpff J, Newick K, Held P, Taatjes D, et al (2013) Mitochondrial-targeted nitroxides disrupt mitochondrial architecture and inhibit expression of peroxiredoxin 3 and FOXM1 in malignant mesothelioma cells. J Cell Physiol 228: 835–845. 10.1002/jcp.24232 23018647 PMC3928986

[57] Cao Z, Bhella D, Lindsay JG (2007) Reconstitution of the mitochondrial PrxIII antioxidant defence pathway: general properties and factors affecting PrxIII activity and oligomeric state. J Mol Biol 372: 1022–1033. 17707404 10.1016/j.jmb.2007.07.018

[58] Cunniff B, Snider GW, Fredette N, Hondal RJ, Heintz NH (2013) A direct and continuous assay for the determination of thioredoxin reductase activity in cell lysates. Anal Biochem 443: 34–40. 10.1016/j.ab.2013.08.013 23973629 PMC3839276

[59] Cunniff B, Snider GW, Fredette N, Stumpff J, Hondal RJ, Heintz NH (2014) Resolution of oxidative stress by thioredoxin reductase: Cysteine versus selenocysteine. Redox Biol 2: 475–484. 10.1016/j.redox.2014.01.021 24624337 PMC3949094

[60] Zhang L, Ging NC, Komoda T, Hanada T, Suzuki T, Watanabe K (2005) Antibiotic susceptibility of mammalian mitochondrial translation. FEBS Lett 579: 6423–6427. 16271719 10.1016/j.febslet.2005.09.103

[61] Yun J, Finkel T (2014) Mitohormesis. Cell Metab. 2014 5 6;19(5):757–66 10.1016/j.cmet.2014.01.011 24561260 PMC4016106

[62] Cunniff B, Wozniak AN, Sweeney P, DeCosta K, Heintz NH (2014) Peroxiredoxin 3 levels regulate a mitochondrial redox setpoint in malignant mesothelioma cells. Redox Biol 3: 79–87. 10.1016/j.redox.2014.11.003 25462069 PMC4297934

[63] Faivre S, Djelloul S, Raymond E (2006) New paradigms in anticancer therapy: targeting multiple signaling pathways with kinase inhibitors. Semin Oncol 33: 407–420. 16890796 10.1053/j.seminoncol.2006.04.005

[64] Xiang X, Phung Y, Feng M, Nagashima K, Zhang J, Broaddus VC, et al (2011) The development and characterization of a human mesothelioma in vitro 3D model to investigate immunotoxin therapy. PLoS One 6: e14640 10.1371/journal.pone.0014640 21305058 PMC3031536

[65] Chappell JBH, R.G. (1972) Preperation and Fractionation In: Birnie GD, editor. Subcellular Components. Butterworths, London.

[66] Zhou M, Diwu Z, Panchuk-Voloshina N, Haugland RP (1997) A stable nonfluorescent derivative of resorufin for the fluorometric determination of trace hydrogen peroxide: applications in detecting the activity of phagocyte NADPH oxidase and other oxidases. Anal Biochem 253: 162–168. 9367498 10.1006/abio.1997.2391

[67] Burch PM, Yuan Z, Loonen A, Heintz NH (2004) An extracellular signal-regulated kinase 1- and 2-dependent program of chromatin trafficking of c-Fos and Fra-1 is required for cyclin D1 expression during cell cycle reentry. Mol Cell Biol 24: 4696–4709. 15143165 10.1128/MCB.24.11.4696-4709.2004 PMC416393

[68] Jonsson TJ, Johnson LC, Lowther WT (2009) Protein engineering of the quaternary sulfiredoxin.peroxiredoxin enzyme.substrate complex reveals the molecular basis for cysteine sulfinic acid phosphorylation. J Biol Chem 284: 33305–33310. 10.1074/jbc.M109.036400 19812042 PMC2785173

[69] Haynes AC, Qian J, Reisz JA, Furdui CM, Lowther WT (2013) Molecular basis for the resistance of human mitochondrial 2-Cys peroxiredoxin 3 to hyperoxidation. J Biol Chem 288: 29714–29723. 10.1074/jbc.M113.473470 24003226 PMC3795269

[70] Poole LB, Godzik A, Nayeem A, Schmitt JD (2000) AhpF can be dissected into two functional units: tandem repeats of two thioredoxin-like folds in the N-terminus mediate electron transfer from the thioredoxin reductase-like C-terminus to AhpC. Biochemistry 39: 6602–6615. 10828978 10.1021/bi000405w

