# Supplementary figures and images for: Disabling Mitochondrial Peroxide Metabolism via Combinatorial Targeting of Peroxiredoxin 3 as an Effective Therapeutic Approach for Malignant Mesothelioma
Source: PLoS One. 2015 May 26;10(5):e0127310. doi: 10.1371/journal.pone.0127310 (PMC4444329; doi:10.1371/journal.pone.0127310)

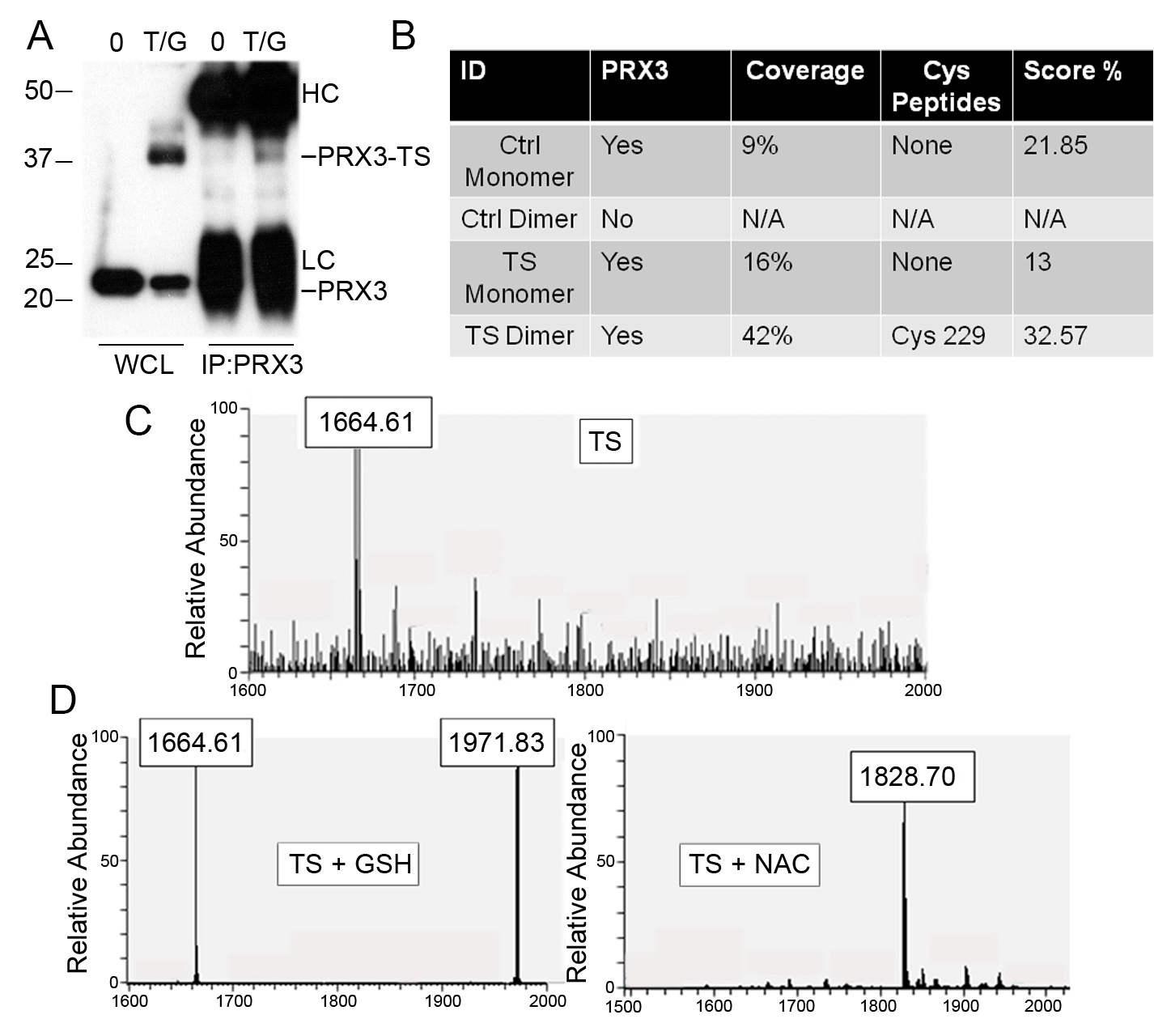

Supplement: S1 Fig — (A) Whole cell lysate (WCL) from HM cells treated with 5 μM TS and 1 μM gentian violet (T/G) were resolved by reducing and denaturing PAGE and immunoblotted for PRX3 (Lanes 1 and 2). PRX3 was immunoprecipitated from 100 μg of total protein from control cell lysates (0) and TS/GV lysates and resolved by reducing and denaturing PAGE (lanes 3 and 4) (B) Bands corresponding to PRX3 monomers (~23 kD) and dimers (~40 kD) were recovered from the gel shown in panel C and digested with trypsin. Peptides corresponding to PRX3 were identified by LC-MS/MS. (C) MS spectrum of thiostrepton (TS, 1664.61 Da). (D) Thiostrepton was incubated with glutathione (GSH, left) or N-acetyl-L-cysteine (NAC, right) in reaction buffer and analyzed by LC–MS. (TIF) [file pone.0127310.s001.tif]

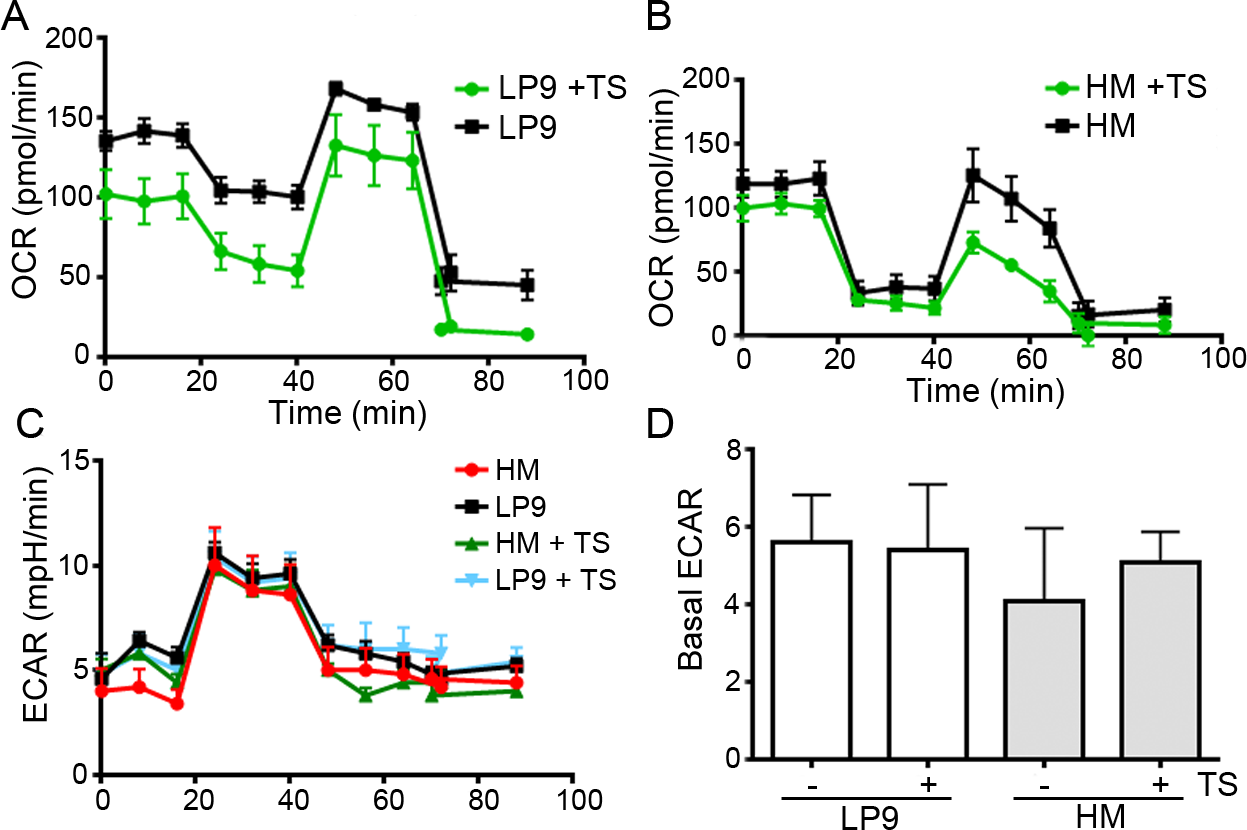

Supplement: S2 Fig — (A) Oxygen consumption rate (OCR) for LP9 cells treated with or without 5 μM thiostrepton (TS) for 6 hrs. (B) OCR for HM cells treated with or without 5 μM TS for 6 hrs. (C) Extracellular acidification rate (ECAR) for LP9 and HM cells treated with or without TS for 6 hrs. (D) Basal ECAR for LP9 and HM cells with or without TS. Error bars represent SEM. (TIF) [file pone.0127310.s002.tif]

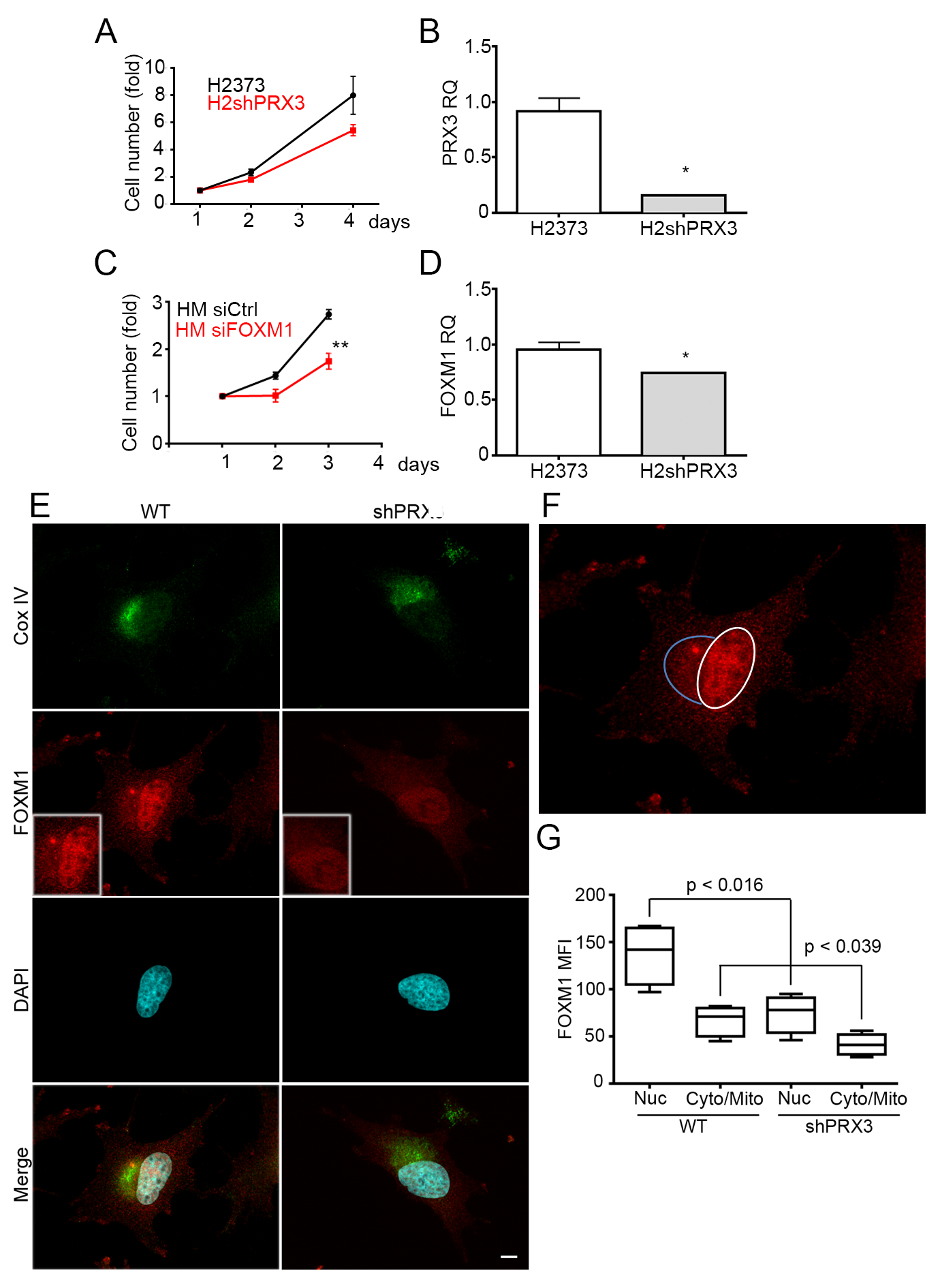

Supplement: S3 Fig — (A) Nuclear staining was used to determine cell number in H2373 cells and shPRX3 H2373 cells (H2shPRX3) over 4 days (n = 4). (B) PRX3 transcript levels in H2373 cells and H2shPRX3 cells (n = 3 * p < 0.05). (C) Nuclear staining was used to determine cell number in HM cells transfected with scramble or FOXM1 siRNA (n = 4, ***p < 0.001). Error bars represent SEM. (D) FOXM1 transcript levels in H2373 cells and H2shPRX3 cells as determined by qRT-PCR (n = 3, * p < 0.05). E) WT and HMshPRX3 cells were fixed and immunostained for FOXM1 and Cox IV (to visualize mitochondrial structures); nuclei were counterstained with DAPI (scale bar = 10 μm). (F) Regions of interest were drawn around the nucleus (Nuc, white circle) and mitochondrial compartment (Cyto/Mito, blue half circle). Mean fluorescence intensity (MFI) is plotted in (G) for representative mitochondrial and nuclear compartments of indicated cell lines (n = 10 cells). Error bars represent SEM. (TIF) [file pone.0127310.s003.tif]

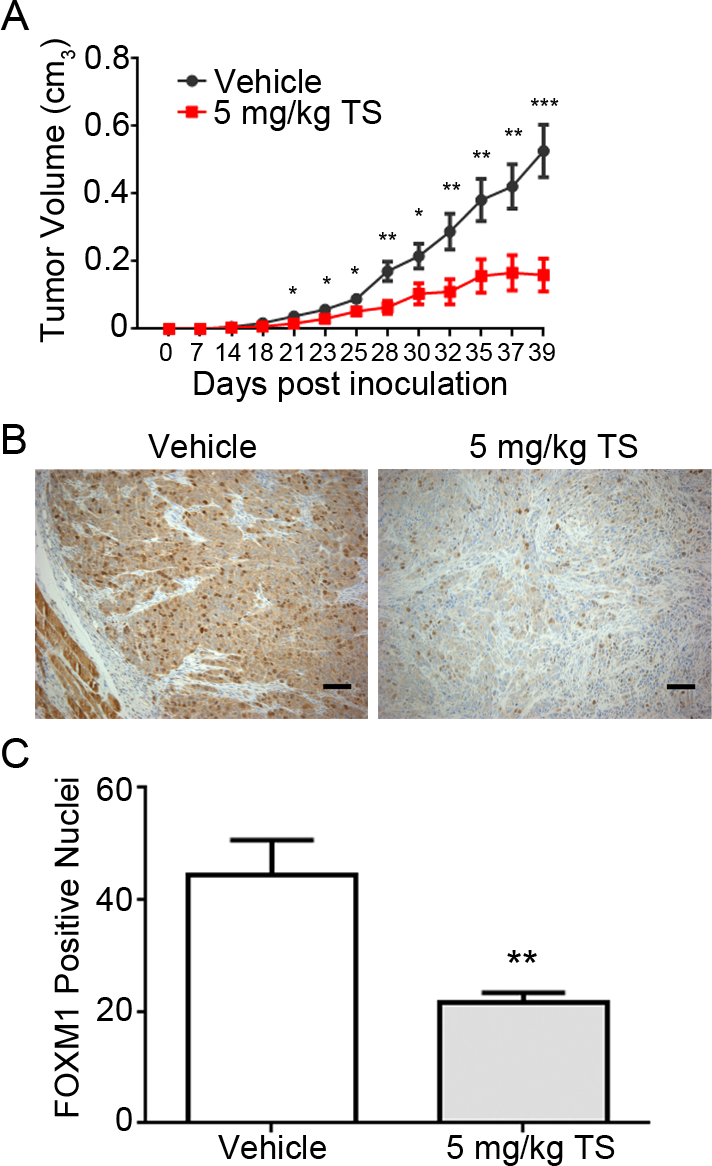

Supplement: S4 Fig — A) Fox Chase SCID mice were injected subcutaneously with HM cells as described in Materials and Methods. After tumors became palpable (about 2 weeks) mice were injected IP with 5 mg/kg TS dissolved in 10% dimethylacetamide (10% DMA) or vehicle control every other day for the indicated number of days. Just prior to each TS injection tumor volume was estimated using calipers. At sacrifice, tumors were dissected and tumor volumes were measured; tumor volume in TS treated animals was significantly different from that of controls (n = 6 mice per group, results shown are representative of 2 independent experiments, ***p < 0.001, *** p < 0.01, * p < 0.05). Analysis of lung and liver specimens revealed no evidence of cytotoxicity due to TS treatment. B) Paraffin-embedded tumor sections were processed for immunohistochemical detection of FOXM1 by IHC (scale bar = 50 μm). C) Nuclear FOXM1 expression was quantified by counting the number of cells with positive nuclear staining in 5 quadrants per section (n = 5, ** p < 0.01). Error bars represent SEM. (TIF) [file pone.0127310.s004.tif]

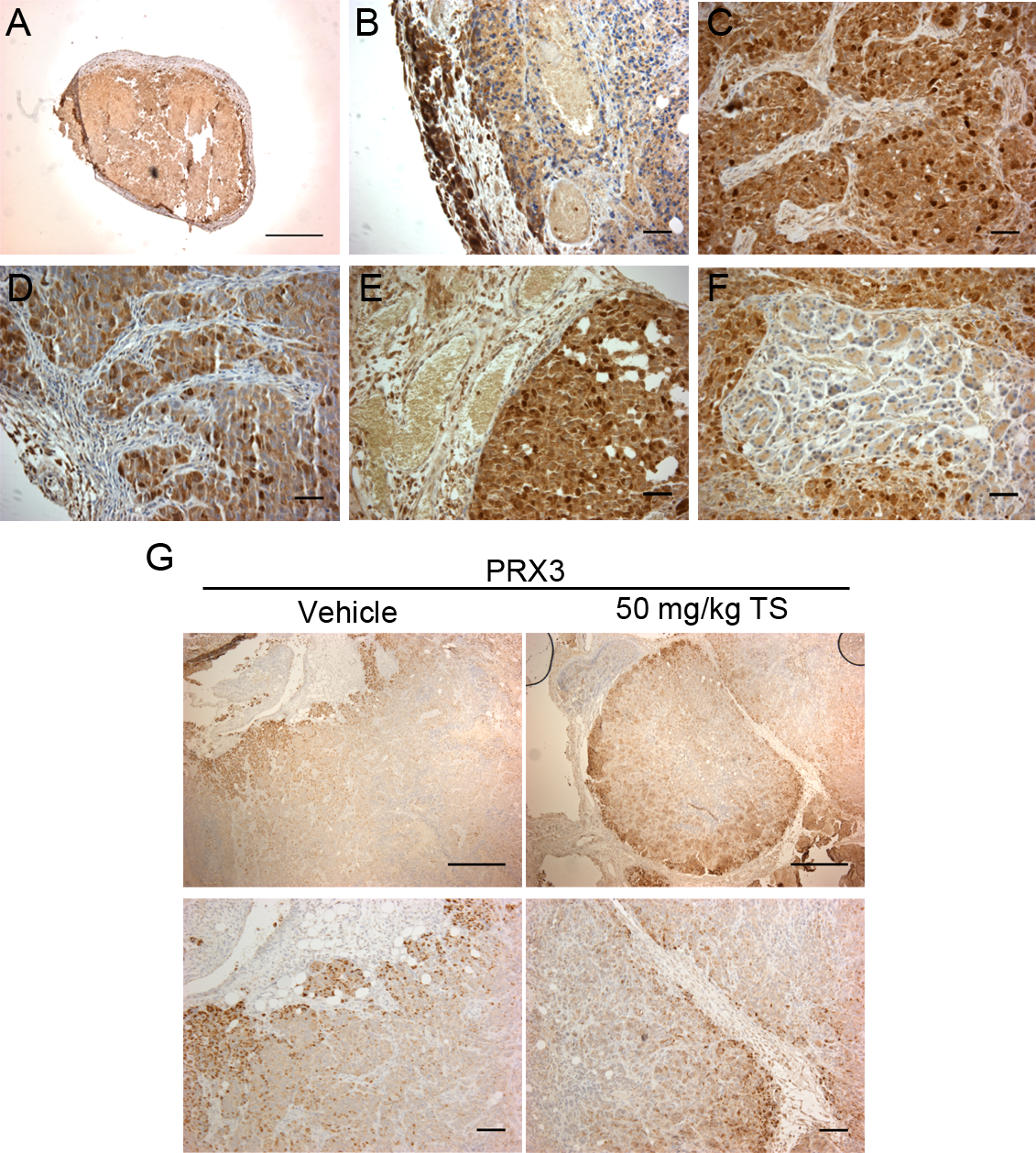

Supplement: S5 Fig — A) Free-floating tumor spheroids measured 3–5 mm in diameter and often contained necrotic areas (scale bar = 0.5 mm). B) Tumor spheroids were typically encapsulated by several layers of FOXM1-positive cells. C and D) FOXM1-positive tumor cells often displayed clear areas between cells, a histological feature of MM due to the presence of microvilli. FOXM1-positive tumor tissue was commonly interspersed with stroma characterized by fibroblastic cells, presumably of mouse origin. E and F) Mesenteric tumors often showed evidence of invasion into abdominal organs such as liver and pancreas (scale bar = 50 μm). G) PRX3 immunohistochemistry staining in vehicle and 50 mg/kg TS tumor sections (scale bar top panels = 0.5 mm, bottom sections = 100 μm). (TIF) [file pone.0127310.s005.tif]
