# Supplementary material for: Disabling Mitochondrial Peroxide Metabolism via Combinatorial Targeting of Peroxiredoxin 3 as an Effective Therapeutic Approach for Malignant Mesothelioma
Source: PLoS One. 2015 May 26;10(5):e0127310. doi: 10.1371/journal.pone.0127310 (PMC4444329; doi:10.1371/journal.pone.0127310)
Supplement: S1 Table — n/o = not observed. (PDF) [file pone.0127310.s006.pdf]

Supplemental Table I.

		Predicted	Obs (control)	$\Delta_{Obs-Pred}$	Obs (TS)	$\Delta_{Obs-Pred}$
Cys peptide						
C108-SH		2976.5	2986.1	+9.6	n/o	
C108-SO_2		3008.5	3017.6	+9.1	n/o	
C127		3495.8	3503.4	+7.6	3502.9	+7.1
C229		3330.7	3339.2	+8.5	3339.2	+8.5
Thiostrepton Cys peptide adduct						
C108-TS		4641.3	4650.5	+9.2	4649.8	+8.5
C127-TS		5160.6	n/o		5164.5	+0.9
C229-TS		4995.5	n/o		4999.8	+1.0
2-Cys disulfide						
C108-C127		6469.3	6468.6	-0.7	6468.5	-0.8
C108-C229		6304.2	6303.4	-0.8	6302.0	-2.2
C127-C229		6823.5	6823.0	-0.5	6822.4	-1.1
Thiostrepton-2 Cys peptide adduct						
C108-TS-C127		8135.1	n/o		n/o	
C108-TS-C229		7971.0	n/o		7970.2	-0.8
C127-TS-C229		8489.3	n/o		n/o	
